# Comparative Hydrodynamic Analysis and Optimization of Gyroid and Diamond Scaffolds with Functionally Graded Porosity

**DOI:** 10.3390/jfb17070320

**Published:** 2026-07-03

**Authors:** Boming Gong, Jia’ao Zhu, Yun Guo, Yameng Xiao, Hongwen Xu

**Affiliations:** School of Mechanical and Automotive Engineering, Shanghai University of Engineering Science, Shanghai 201620, China; m310124503@sues.edu.cn (B.G.); 251210008@st.usst.edu.cn (J.Z.); m310124501@sues.edu.cn (Y.X.); xhw194201@163.com (H.X.)

**Keywords:** bone tissue engineering, TPMS structure, Gyroid, porosity gradient, computational fluid dynamics

## Abstract

This study presents a numerical investigation into the hydrodynamic and biomechanical performance of bone-repair scaffolds based on Triply Periodic Minimal Surfaces (TPMSs). Focusing on Gyroid and Diamond architectures, scaffolds with uniform (40–70%) and functionally graded porosities were developed. Computational Fluid Dynamics (CFD) simulations were employed to evaluate permeability, pressure drop, and Wall Shear Stress (WSS) distributions. Results indicate distinct topological advantages: the Gyroid structure demonstrates superior permeability and uniform WSS distribution due to its isotropic fluid channels, whereas the Diamond structure maintains better flow velocity stability. Crucially, the introduction of a porosity gradient (40–60%) successfully mitigates localized pressure surges and optimizes the bioactive WSS window for cell differentiation. Notably, increasing porosity to 70% in Gyroid scaffolds yielded a 277% enhancement in permeability. These findings establish a theoretical basis for designing functionally graded TPMS scaffolds that balance fluid transport efficiency with a favorable cellular microenvironment.

## 1. Introduction

With the increasing aging of the population and the rising incidence of bone defects, higher clinical demands are being placed on bone-repair materials, particularly regarding mechanical adaptability, biocompatibility, and regenerative capability [1]. Although traditional bone-repair methods, such as autografts and allografts, have achieved certain success in treating bone defects [2,3,4,5], they still face a series of challenges, including donor shortage, immune rejection, and risks associated with secondary surgeries. Especially when the bone defect exceeds the critical threshold (typically over 2 cm) [6,7,8], traditional approaches struggle to meet repair demands. Therefore, there is an urgent need to develop novel bone-repair materials and methods to enhance healing efficacy and overcome the limitations of existing techniques. To address the shortage of bone resources and alleviate the reliance on bone grafts in traditional treatments, bone-tissue engineering (BTE) has emerged as a promising field [9,10,11]. It leverages biocompatible materials and engineering techniques to develop functional bone scaffolds. In recent years, scaffold designs based on triply periodic minimal surface (TPMS) structures have increasingly become a research hotspot. Due to their superior mechanical properties, biomimetic features, and high specific surface areas, TPMS structures exhibit great potential in BTE [12,13,14]. To promote cell growth, bone-repair scaffolds must account for nutrient transport requirements, ensuring the interconnectivity of porous channels for cell migration and favorable surface conditions for cell attachment, including surface roughness, chemical functional group distribution, and topological morphology. To evaluate these properties, permeability is commonly employed as a key metric; higher permeability facilitates cell infiltration into the interior of the porous material and enhances the diffusion efficiency of nutrients, particularly macromolecular growth factors and oxygen, thereby creating more favorable conditions for cell growth. Concurrently, wall shear stress (WSS) is another critical parameter. Studies have shown that different levels of WSS exert distinct mechanical stimuli on mesenchymal cells, leading to variations in the cell differentiation process [15,16]. Compared to traditional scaffold designs, TPMS features large-scale and continuous concave surfaces with subtle curvature variations. This characteristic mimics the microstructure of natural bone tissue, promoting cell proliferation and differentiation on TPMS scaffolds.

Numerical simulation can be utilized to analyze the performance of bone-repair materials, allowing researchers to evaluate various metrics before fabrication, including the time-dependent relationship between dynamic degradation behavior and mechanical properties. Guided by the simulation results, targeted measures can be adopted—such as altering the scaffold material or its geometric characteristics (e.g., wall thickness, strut angles, or gradient porosity distributions)—to regulate the overall performance without the need to remanufacture the entire structure, thereby reducing the costs associated with producing new scaffolds. One of the most critical simulation methods in BTE is computational fluid dynamics (CFD). Through CFD, fluid flow through the scaffold can be analyzed to examine the pressure distribution (revealing local flow stagnation zones), permeability (quantifying channel interconnectivity efficiency), specific surface area (evaluating cell attachment potential), fluid velocity (predicting nutrient concentration gradients), and WSS levels (reflecting the spatial distribution of mechanical stimuli). This enables a better understanding of how the specific geometry of each scaffold influences the cell growth process [17,18]. Furthermore, besides analyzing scaffold performance, computer simulations can also be applied to optimize scaffold geometries to modulate Young’s modulus [19], compressive strength [20], or octahedral shear strain [21,22,23]. Nevertheless, current optimization strategies in BTE focus almost exclusively on the mechanical performance of the scaffold itself, with less emphasis placed on the internal fluid flow, thereby leaving the fluid–cell interactions insufficiently explored.

In previous studies, Germain et al. [24] fabricated a biodegradable TPMS scaffold using PLA via FDM technology, which exhibited good mechanical properties, biocompatibility, and degradation behavior. Li et al. [12] 3D printed and evaluated a TPMS-based graded scaffold in vivo for treating segmental bone defects; the finite element method (FEM) analysis clearly demonstrated that this structure provided a more uniform stress distribution and supported favorable cell proliferation. Despite the immense potential that TPMS scaffolds offer in BTE, due to their high specific surface area and exceptional geometric interconnectivity, current research remains largely confined to homogeneous structures with a single porosity. This design paradigm often struggles to reconcile the inherent contradiction between mechanical support and biological permeability, resulting in noticeable limitations when simulating the complex mechanical environment of natural bone. Structurally, natural bone tissue exhibits significant spatial heterogeneity: the highly dense, low-porosity cortical bone bears the primary mechanical loads, while the highly porous trabecular bone provides an ideal environment for bone marrow support and metabolic exchange. Traditional uniform TPMS structures, lacking such hierarchical gradients, are prone to inducing stress shielding effects and may restrict the deep vascularization process, due to impaired nutrient delivery in the central region.

Despite the growing body of literature on TPMS scaffolds, a critical gap persists: no study has systematically compared the hydrodynamic performance of Gyroid and Diamond architectures under a controlled, linear porosity gradient via CFD simulation. Existing investigations have focused either on a single TPMS topology across uniform porosity levels, or have incorporated gradients without isolating the role of architectural topology. In particular, the interplay between the isotropic curved channels of the Gyroid and the angular-channeled Diamond structure—when subjected to an identical linear porosity gradient from 40% to 60%—remains uncharacterized. Moreover, most gradient studies report only mechanical properties, leaving the WSS distribution and permeability implications of functionally graded designs largely unexplored [25]. The present work addresses these gaps by (1) providing a systematic, head-to-head CFD comparison of Gyroid and Diamond TPMS scaffolds across both uniform (40–70%) and linearly graded (40–60%) porosities; (2) quantifying the topology-specific response to a linear porosity gradient in terms of permeability, pressure drop, and WSS distribution; and (3) establishing design guidelines for selecting the appropriate TPMS topology and gradient strategy based on targeted biomechanical performance criteria.

Therefore, to address the demand for biomechanical performance optimization of bone-tissue repair scaffolds, this study combines triply periodic minimal surface (TPMS) structural designs with functional grading strategies. Through numerical simulation techniques, the hydrodynamic characteristics and cellular microenvironment regulation mechanisms of porous bone-repair scaffolds are systematically analyzed. An optimized design methodology for biomimetic gradient scaffolds is proposed, providing a theoretical basis for the personalized design of bone defect repair materials.

## 2. Materials and Methods

### 2.1. TPMS Scaffold Modeling

In this study, sheet-like TPMS bone-repair scaffolds were constructed using the iso-surface offsetting method. This method defines the solid domain as the closed region between two isosurfaces with equal magnitudes but opposite directions—namely, level-set constants (e.g., c1 = c0 + Δc and c2 = c0 − Δc)—via a dual level-set thresholding strategy. This design enables a shell topological configuration with uniform wall thickness, where the relative density is directly regulated by the offset amount Δc, overcoming the dependence of traditional grid-like structures on topological interconnectivity thresholds. The selection of a sheet-like design was primarily based on the following technical advantages:

Structural integrity at low densities: traditional grid-like structures are prone to loss of connectivity due to necking fracture at low volume fractions (e.g., below 8%). In contrast, by independently controlling the wall thickness, the sheet-like structure maintains excellent structural integrity and functional adaptability within a low relative density range of 5–30%.

Superior hydrodynamic and biological properties: the sheet-like structure features continuous and smooth surfaces, significantly reducing flow resistance and enhancing the specific surface area. Taking the Gyroid configuration as an example, its flow resistance coefficient can be reduced by 40–60%, compared to grid-like structures of the same density, and its specific surface area increases by approximately 1.8 times, which greatly promotes osteoblast attachment and nutrient diffusion.

Progressive energy absorption: during mechanical loading, sheet-like TPMS can achieve an energy dissipation efficiency of up to 85% through the progressive buckling deformation of the curved surfaces, effectively preventing structural failure caused by brittle node collapse.

Regarding configuration selection, this study primarily focused on two typical TPMS structures: Gyroid and Diamond [26,27]. The Diamond structure, with its exceptionally high stiffness-to-weight ratio and excellent self-supporting angles, significantly improves forming accuracy during additive manufacturing processes (such as SLM/SLA).

**Figure 1:**
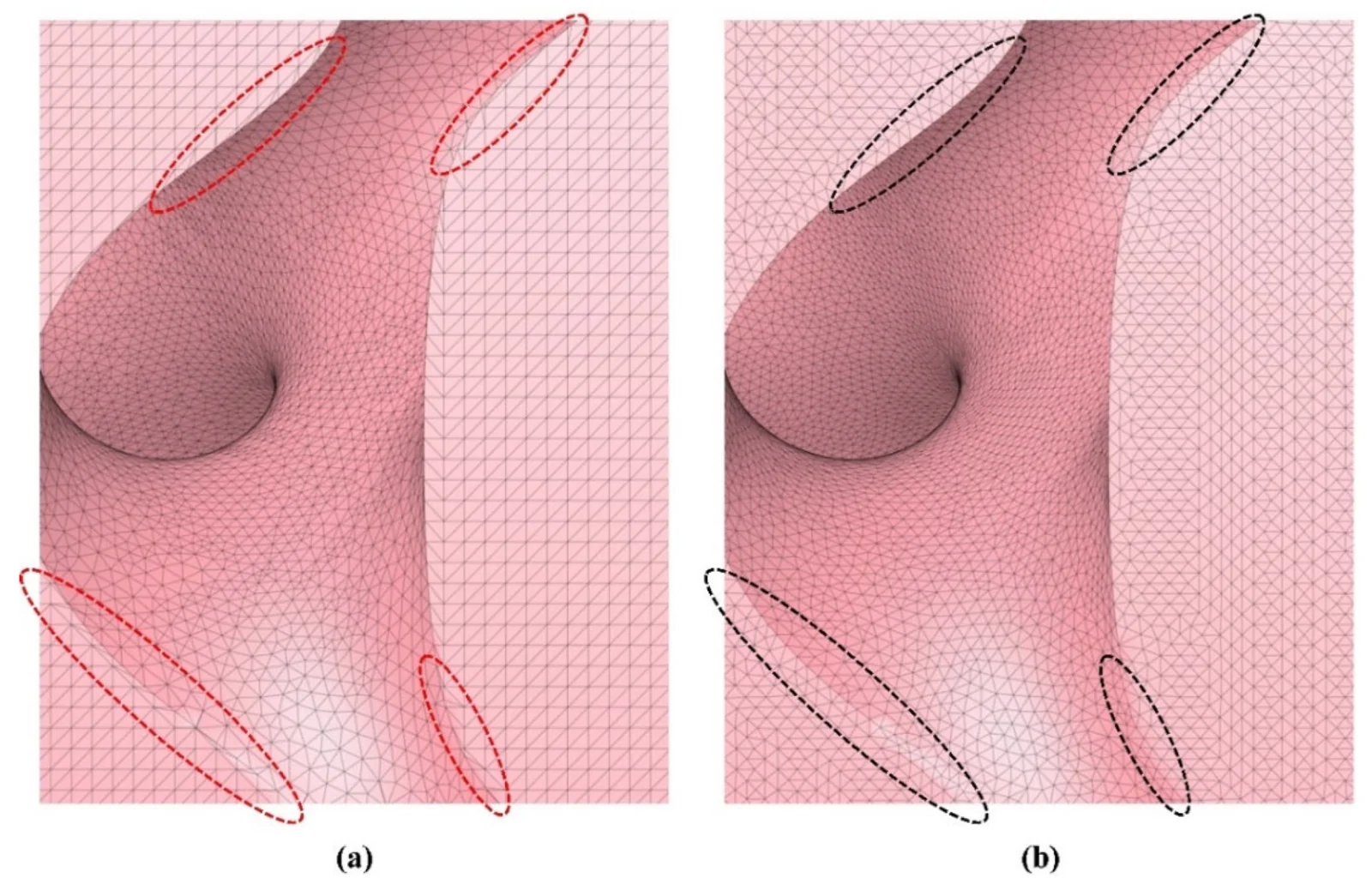
Mesh repair results. (**a**) Mesh directly output by Mathematica; (**b**) re-meshed by Fusion.

Due to the complex continuous curved surfaces of TPMS structures, Wolfram Mathematica 14 was utilized in this study to generate the TPMS lattice models. The generated STL files may contain facets that are overly dense or sparse in boundary regions. To prevent this from affecting simulation accuracy, Autodesk Fusion (v.2.0.19994, Autodesk Inc., San Rafael, CA, USA) was used for mesh optimization, and Magics 24.0 was employed to repair potential geometric defects, such as sharp triangular facets and overlapping shells, ensuring smooth surfaces and high geometric fidelity. As illustrated in Figure 1a, the red circle marks the region at the edge of the structure with sharp triangular facets. Figure 1b demonstrates that after re-meshing with Fusion, the sharp triangular facets in the same region at the edge of the scaffold are significantly reduced.

**Figure 2:**
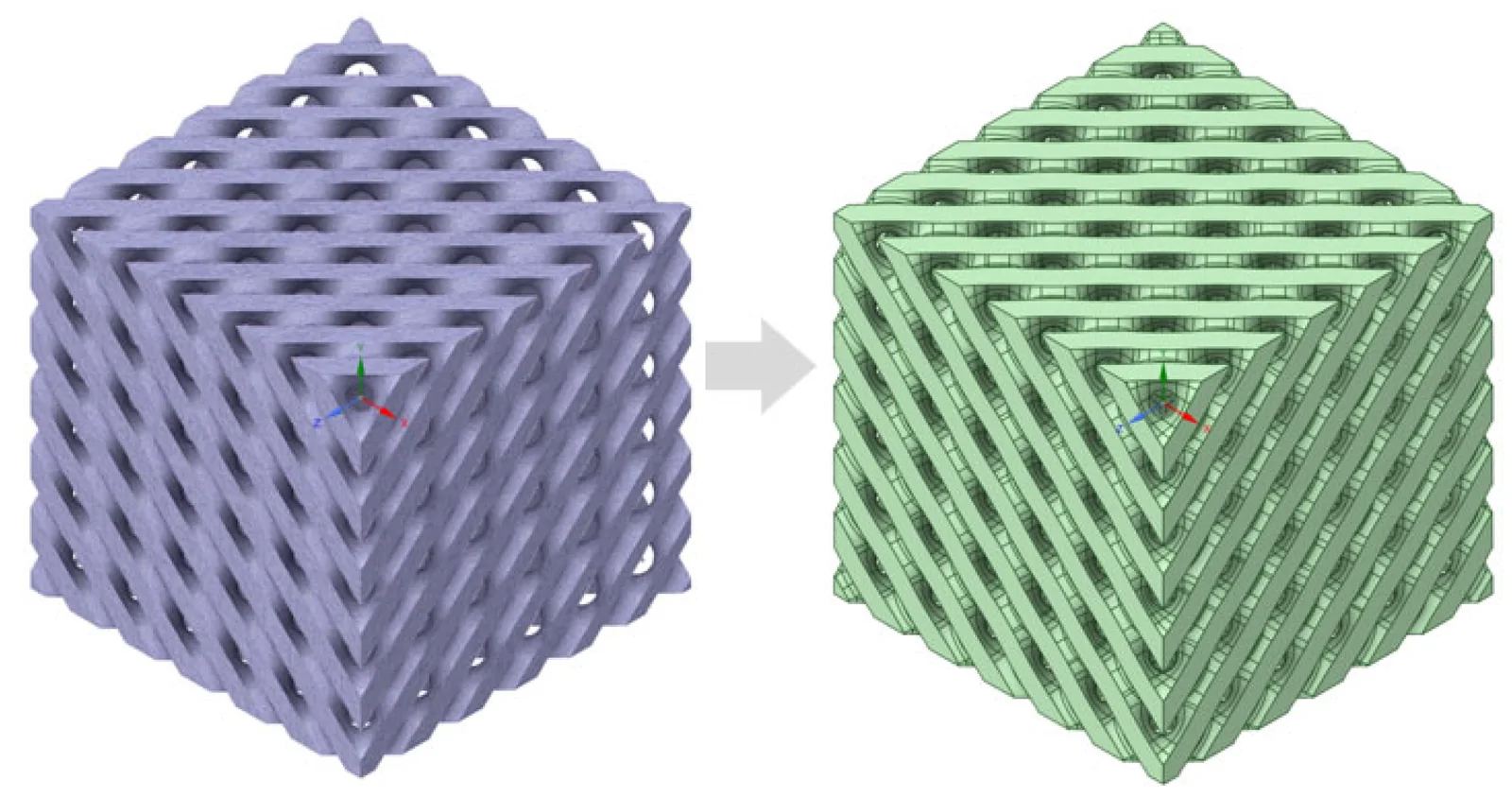
Schematic of the solidification process for the diamond lattice scaffold.

The processed STL file was transformed into a 3D solid and solidified using SpaceClaim within Ansys Workbench (2024 R2, Ansys Inc., Canonsburg, PA, USA) to facilitate subsequent fluid simulation and analysis. During this process, 3D reconstruction ensured the integrity of the internal volume information of the scaffold, providing an accurate geometric basis for fluid flow simulations. Figure 2 presents a schematic diagram of a Diamond lattice scaffold transitioning from triangular facets in STL format (left) to a 3D solid (right). This completes the preprocessing of the TPMS lattice scaffold.

### 2.2. Porosity Gradient Grading

**Figure 3:**
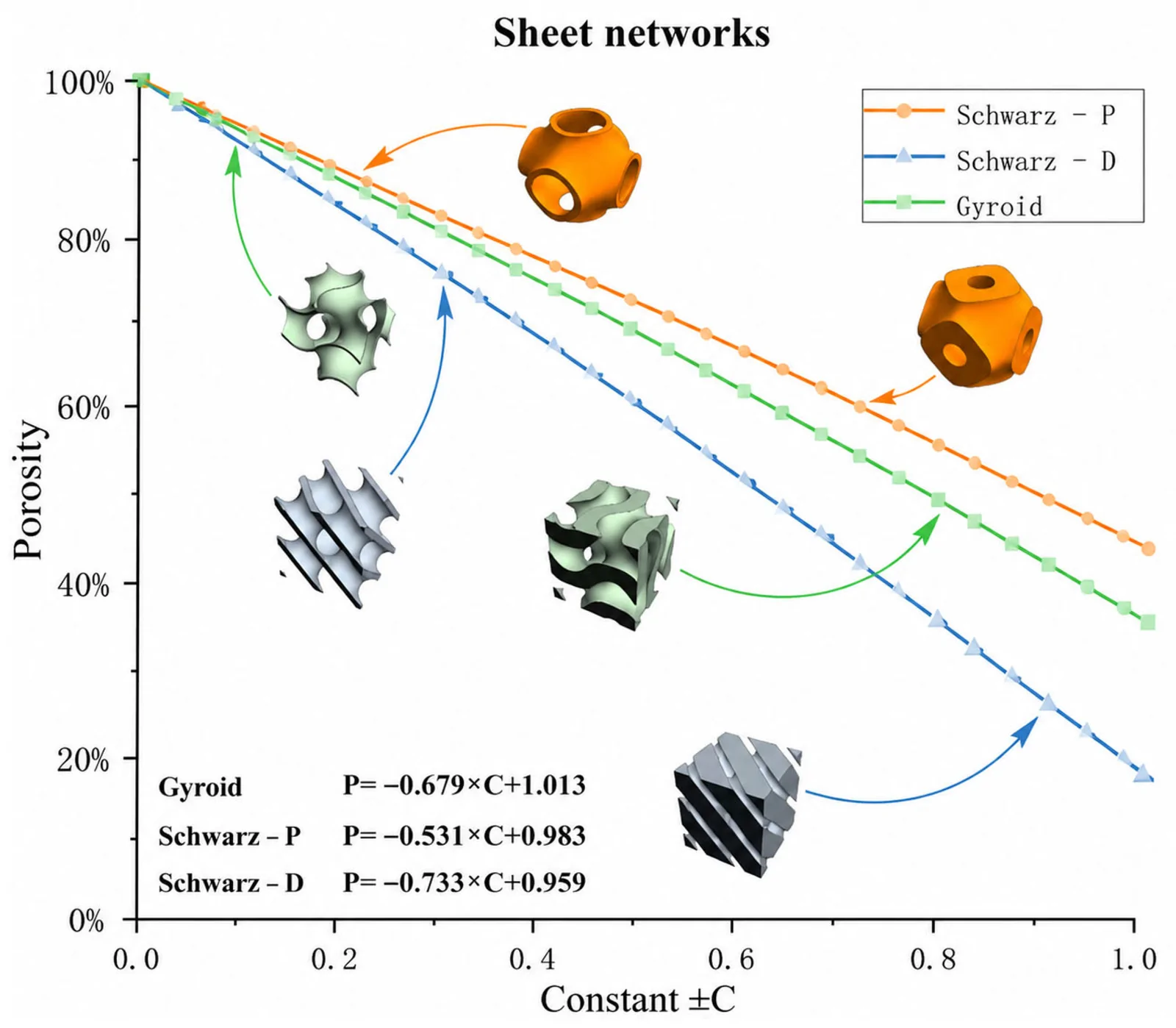
The porosity variation of sheet-network structures in different TPMS type with respect to the level-set parameter c.

Functionally graded bone-repair scaffolds with porosity grading can simulate the porosity variance of natural bone from trabecular to cortical bone to accommodate the biomechanical demands of different regions. Trabecular bone possesses a higher porosity, providing a lighter structure and superior bone marrow support, while cortical bone features smaller pores and offers stronger mechanical support. Through a porosity grading design, functionally graded scaffolds not only resemble natural bone in mechanical properties, but also promote bone-tissue growth and repair at different levels, enhancing vascularization and cell adhesion, thereby treating bone defects more effectively and achieving more natural bone-tissue regeneration. Porosity can be functionally graded by altering the spatial values of the level parameter in Cartesian space, where the spatial value of constant c varies, according to a certain function or tabular data. Before initiating the design of gradient porosity scaffolds, it is first necessary to acquire the relationship between the lattice porosity and the level parameter, C. In this study, Gyroid, Schwarz-P, and Diamond TPMS configurations were selected. In Wolfram Mathematica, 40 points were uniformly sampled for the level-set parameter C between 0 and 1, via source code. Subsequently, this point set was input into a packaged porosity calculation code. By altering the input TPMS level-set formulas, the variation of the sheet-like lattice porosity with parameter C was obtained for different configurations. The measured data were linearly fitted to yield Figure 3. Here, the Schwarz-P configuration was included alongside Gyroid and Diamond, strictly to validate the universal applicability of the linear thresholding methodology across different primitive TPMS mathematical families. However, since the primary objective of this study is to systematically evaluate the synergistic effects of continuous porosity gradients on transport performance, the subsequent CFD simulations and biomechanical characterizations focus exclusively on the Gyroid and Diamond architectures due to their superior flow connectivity and morphological advantages in bone-tissue engineering.

Based on the relationship between the porosity and level parameter C of different TPMS lattices, the porosity distribution of the lattice scaffold can be flexibly adjusted. For instance, to achieve a sheet-like Diamond configuration with porosity varying from 50% to 90% along the positive *z*-axis, c is constructed as a linear function of z. The sheet-like Diamond structure corresponds to a c value of 0.6262 at 50% porosity and 0.0805 at 90% porosity. Assuming the scaffold dimensions are 5 mm × 5 mm × 15 mm, with a single period in the x and y directions and 3 repeating periods in the z direction (arranged as 1 × 1 × 3), the 3D dimensions of a single unit cell become 5 mm × 5 mm × 5 mm. Let z = 15 correspond to 50% porosity and z = 0 to 90%. Then, the level-set parameter describing this porosity gradient variation is c = 0.0364z + 0.0805, and the corresponding overall functional expression for the Diamond sheet structure is

$$-(0.0364z+0.0805)\leq \emptyset_{D}(x,\;y,\;z)\;\leq 0.0364z+0.0805$$

$$\emptyset_{D}(x,y,z)\equiv sin\frac{\;2\alpha \pi \;x}{l1}\;sin\frac{\;2\alpha \pi \;y}{l2}sin\frac{\;2\alpha \pi \;z}{l3}+sin\frac{\;2\alpha \pi \;x}{l1}cos\frac{\;2\alpha \pi \;y}{l2}cos\frac{\;2\alpha \pi \;z}{l3}+cos\frac{\;2\alpha \pi \;x}{l1}sin\frac{\;2\alpha \pi \;y}{l2}cos\frac{\;2\alpha \pi \;z}{l3}+cos\frac{\;2\alpha \pi \;x}{l1}cos\frac{\;2\alpha \pi \;y}{l2}sin\frac{\;2\alpha \pi \;z}{l3}$$

where domain parameters are defined appropriately, and the domain is x ∈ (0,5), y ∈ (0,5), z ∈ (0,15).

**Figure 4:**
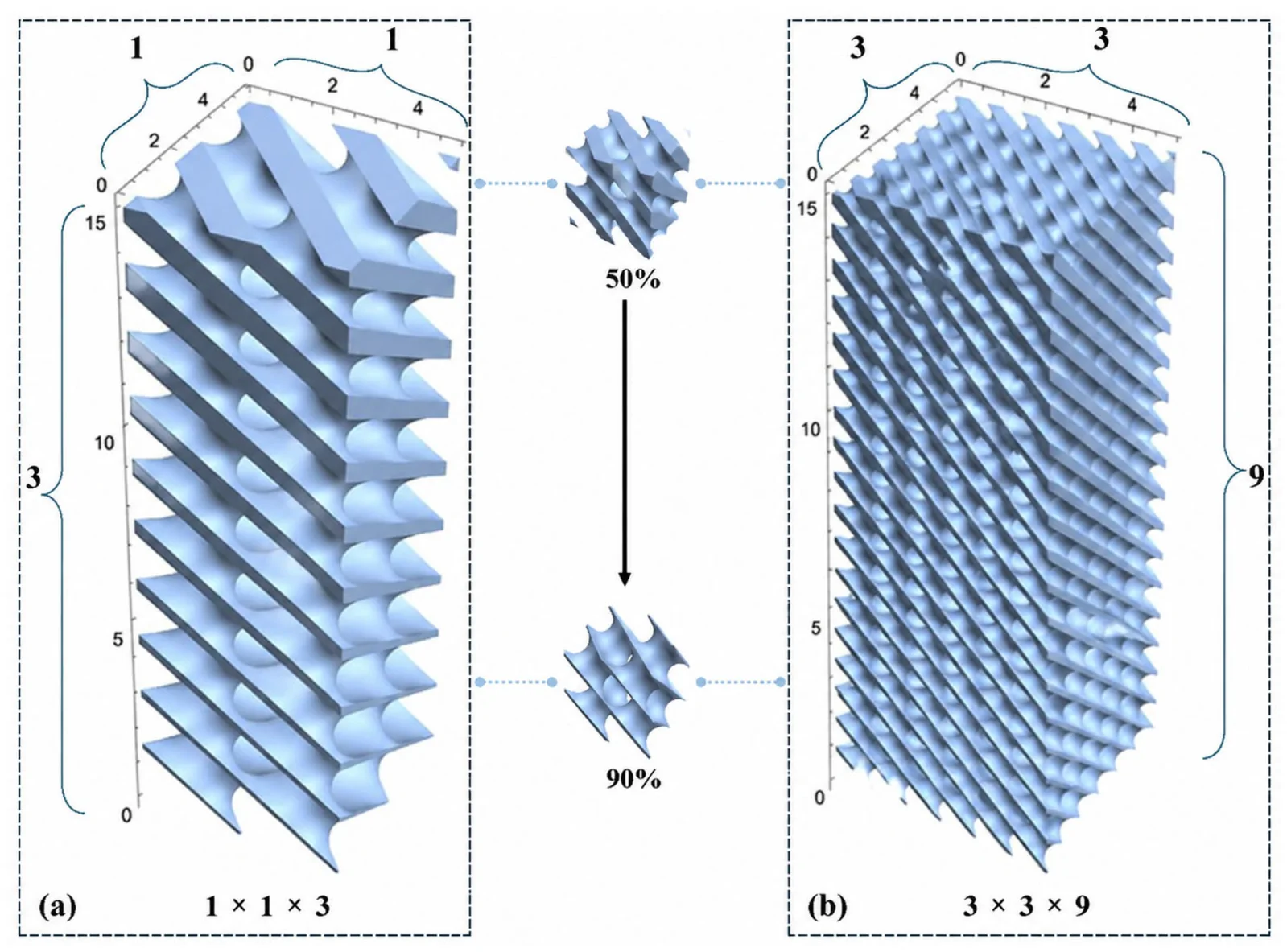
(**a**) Diamond sheet network scaffold (1 × 1 × 3 array) with graded porosity from 50% to 90%; (**b**) Diamond sheet network scaffold (3 × 3 × 9 array) with graded porosity from 50% to 90%.

Figure 4a presents a schematic of a sheet-like Diamond lattice scaffold with porosity graded from 50% to 90%. Similarly, the topological structure of the scaffold can be further designed; for example, by setting 3 periods of unit cells in the x and y axes and 9 periods in the z axis, the length, width, and height dimensions of each unit cell are controlled at 1.67 mm, as shown in Figure 4b.

### 2.3. Numerical Simulation

Upon completion of geometric model preprocessing, spatial discretization was executed. Due to the high-curvature, multi-porous, and complex topological features of TPMS lattice scaffolds, this study utilizes Fluent Meshing 2024 R2 (Ansys Inc., Canonsburg, PA, USA) for discretization. This tool efficiently processes complex geometries and achieves a seamless transition from surface mesh to volume mesh.

To address the complex cavity structures of the scaffolds, this study uniformly employs polyhedral mesh for volume filling. Compared to traditional tetrahedral meshes, polyhedral meshes reduce the total number of elements while achieving higher computational accuracy and significantly suppressing numerical diffusion by increasing the number of contact faces between adjacent elements.

Mesh quality directly dictates the convergence and reliability of numerical solutions. In this study, orthogonal quality and skewness were adopted as the primary evaluation metrics. To ensure computational robustness, the evaluation criteria for all models were set as follows:

Orthogonal Quality: Minimum value not less than 0.15.

Skewness: Maximum value controlled below 0.90.

For local regions with rapid flow variations, such as the interior pores and near-wall regions of the scaffold, local refinement was performed. This non-uniform discretization strategy optimizes the allocation of computational resources while meeting precision requirements. The generated mesh model was imported into the solver for calculation after quality verification.

Upon entering the numerical computation phase, the core task was to configure the computational fluid dynamics (CFD) software environment and execute the solver. In this study, low-speed incompressible flow conditions were simulated, and a pressure-based solver was selected, which couples the velocity and pressure fields by alternately solving the momentum and continuity equations. Specifically, the system first calculates the fluid velocity distribution using the momentum equation, combines mass conservation to calculate the pressure field, and updates the velocity and pressure fields through an iterative process until residuals meet predefined convergence criteria. Additionally, to accurately simulate fluid motion under gravity, gravitational acceleration along the *Z*-axis was set to −9.81 m/s^2^.

The numerical simulation analyzed critical biomechanical parameters of the bone-repair scaffolds, such as permeability, wall shear stress (WSS), and specific surface area, to evaluate their influence on cell proliferation and differentiation. Permeability characterizes the resistance of the material to fluid flow. Specifically, it reflects the fluid volume passing through a unit area per unit time under a unit pressure difference, typically expressed by Darcy’s law:

(1) $$k=\frac{Q\cdot \mu \cdot L}{A\cdot ∆P},$$

where *Q* is fluid flow rate (m^3^/s), μ is dynamic viscosity (kg/m ·s ), *L* is model length (m), *A* is cross-sectional area at the inlet (m^2^), and ∆P is pressure drop (Pa).

Specific Surface Area (SSA) is defined as the total surface area per unit mass or volume of the material, calculated as follows:

(2) $$s=\frac{S}{V_{s}},$$

where *s* is specific surface area, *S* is surface area, and $V_{s}$ is the volume of the scaffold solid portion.

Wall shear stress can be calculated by the formula

(3) $$\tau_{w}=\eta \frac{\partial u}{\partial n},$$

where η is dynamic viscosity (Pa · s), *u* is fluid velocity (m/s), and *n* is the direction normal to the wall.

Permeability should ideally approach the average value of natural trabecular bone (5.13 × 10^−9^ m^2^, range 0.02 × 10^−9^ m^2^ to 20 × 10^−9^ m^2^) to prevent mechanical instability or cellular activity inhibition caused by overly high or low values [28,29,30]. The ideal range of WSS is 0.1–10 mPa [31,32], which promotes osteogenic differentiation. Quantitative analysis of these parameters provides a basis for scaffold optimization.

Before initiating the simulation to determine the flow regime, the Reynolds number (*Re*) was calculated using the following expression:

(4) $$Re=\frac{\rho uL}{\mu },$$

where *ρ* is fluid density (kg/m^3^), u denotes the characteristic flow velocity(m/s), *L* is characteristic length (m), and *μ* is dynamic viscosity (kg/(m·s)).

Computations confirmed that the Reynolds number Re in this study was 0.0211, which is far less than 1 and significantly below the critical threshold of 2000, indicating laminar flow conditions. Thus, a laminar flow model was selected in the settings.

**Figure 5:**
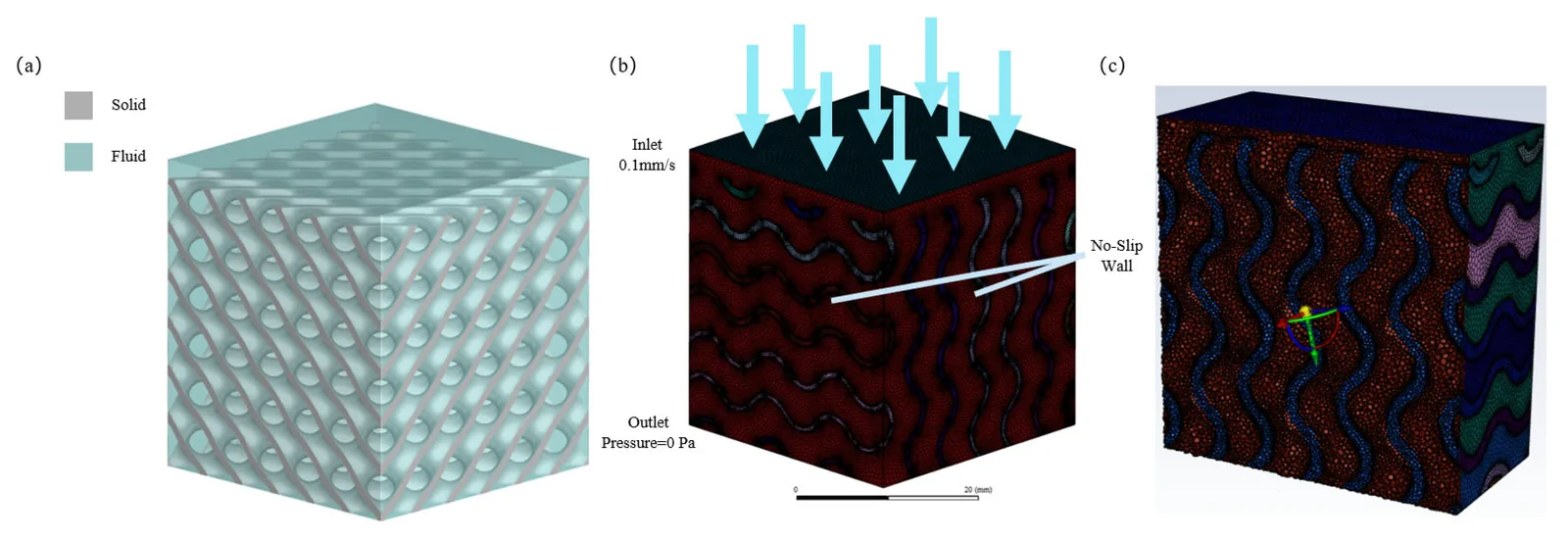
Boundary conditions and meshing. (**a**) Schematic diagram of the fluid and solid domains of the support; (**b**) schematic diagram of the boundary conditions and mesh model; (**c**) grid section.

The fluid adopted the physical properties of Dulbecco’s Modified Eagle Medium (DMEM) [18,33], which contains high concentrations of glucose, amino acids, and buffer systems to provide the necessary metabolic environment for cell proliferation and bone matrix mineralization. Its density is 1000 kg/m^3^, specific heat Cp = 4182 J/(kg·K), thermal conductivity k = 0.6W/(m·K), and dynamic viscosity 0.00145 kg/(m·s) [34,35,36]. The scaffold material is TC4 (Ti-6Al-4V), widely used in bone repair due to its excellent biocompatibility and mechanical strength. Its density is 4430 kg/m^3^, specific heat Cp = 613 J/(kg·K), and thermal conductivity k = 6.93 W/(m·K). The fluid domain boundaries were set as no-slip walls. The boundary conditions and operating parameters of the model are illustrated in Figure 5. Specifically, the schematic representation of the fluid and solid domains of the scaffold is depicted in Figure 5a; the detailed boundary condition settings and the global mesh model are presented in Figure 5b,c, showing the mesh cross-sectional details of the internal pores within the scaffold. The fluid inlet was set to a velocity inlet boundary condition with a velocity of 0.1 mm/s and a temperature of 310 K. The outlet was set to 0 Pa pressure.

In the numerical solution process, the Coupled solver was employed for flow analysis, which simultaneously solves the momentum and pressure-based continuity equations. Although the resource consumption per step is slightly higher, it offers faster convergence and stronger stability in handling low-speed complex flows. Gradient calculations used the Least Squares Cell-Based method. The pressure term adopted the PRESTO! format. Momentum and energy terms used the second-order upwind scheme. The residual convergence criterion was set to 1 × 10^−5^, combined with dynamic under-relaxation factors. Iteration was stopped after ensuring calculation accuracy (i.e., residuals dropped below the threshold and inlet–outlet flow rates were conserved).

### 2.4. Lattice Structures

The topological design of bone-repair scaffolds has primarily evolved from traditional non-parametric topologies to advanced parametric topologies. Early non-parametric designs mainly include honeycomb, circular, and cubic structures. Although honeycomb structures offer the advantages of high stiffness and light weight, they are highly susceptible to multi-directional failure under complex loads. While circular pore structures effectively mitigate stress concentrations, the high porosity (70–90%) [37] required to achieve ideal strength often leads to pore clogging, thereby hindering nutrient transport. Furthermore, although cubic structures exhibit high macroscopic mechanical strength, their stretch-dominated topologies render the struts parallel to the loading direction prone to buckling under load, initiating progressive layer-by-layer catastrophic failure.

To overcome the geometric limitations of these non-parametric designs, parametric design significantly enhances the controllability of pore architectures by introducing adjustable variables. Among these, the Voronoi structure utilizes spatially random discrete points to closely simulate the anisotropic characteristics of natural trabecular bone; however, severe stress concentrations inevitably remain at traditional strut intersections. In contrast, Triply Periodic Minimal Surfaces (TPMSs)—a class of periodic continuous surfaces with zero mean curvature—achieve an exceptionally uniform stress distribution, significantly mitigating the risk of nodal stress concentration. In recent years, the application of TPMS structures in bone-tissue engineering has seen significant progress. For example, Yan et al. [38] fabricated high-porosity (80–95%) scaffolds based on Diamond and Gyroid structures using Ti-6Al-4V, demonstrating an elastic modulus range (0.12–1.25 GPa) comparable to that of natural trabecular bone. Research by Keshavarzan [39] indicated that in compression tests, the P-type TPMS structure exhibited a progressive layer-by-layer failure mode, demonstrating superior cyclic compressive endurance and an extended failure cycle. Notably, the Gyroid structure exhibits even higher flexibility in parametric modulation, allowing it to adapt to diverse mechanical performance demands [40]. Moreover, the fully interconnected micro-channels and high specific surface areas of TPMS structures (such as Gyroid and Diamond) significantly optimize fluid dynamics and mass transport properties, establishing them as the ideal topological choices for fabricating highly biomimetic, functionally graded bone-repair scaffolds via additive manufacturing technologies.

## 3. Results and Discussion

### 3.1. Grid Independence Test

**Figure 6:**
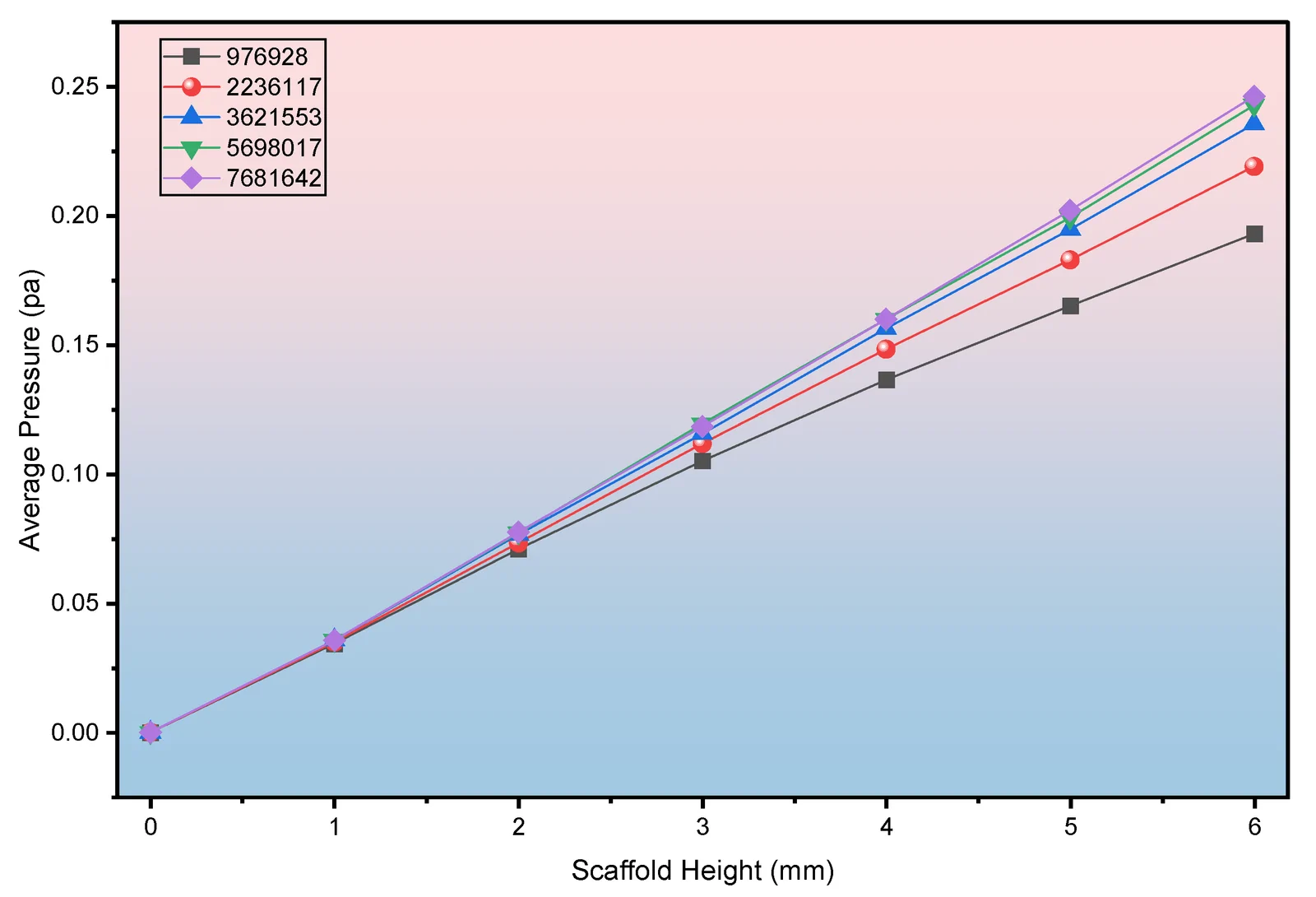
Grid independence test.

To ensure the accuracy of the numerical simulation results, this study generated mesh models with different element numbers by altering the minimum mesh size of the model. The average pressures at different heights of the scaffold corresponding to five groups of different mesh numbers were calculated under the same conditions, as shown in Figure 6 below. Taking the sheet-like unit-cell scaffold model with 60% porosity Gyroid (denoted as 60 G) as an example, the 60 G model was arranged in a 3 × 3 × 3 format with 3D dimensions of 6 mm × 6 mm × 6 mm. To ensure smooth fluid flow, a distance of 0.2 mm was reserved between the top surface of the scaffold and the fluid inlet. Polyhedral mesh models with different element numbers were generated by changing the minimum mesh size of the model, and simulations were performed under the conditions of a fluid inlet velocity of 0.1 mm/s, an outlet pressure of 0 Pa, and a temperature of 310 K. The results indicate that when the number of mesh elements exceeds 3.62 million, the simulation results tend to stabilize. Therefore, 5.69 million mesh elements were selected for the final calculation. To ensure the accuracy of the calculation results, the number of mesh elements should be greater than 3 million. By comparing the average pressure results of the five groups with different mesh numbers at different heights, it is easy to find from the figure that the difference between the two groups with mesh element numbers of 0.97 million and 2.23 million is the most significant, with a maximum difference of up to 13.25%. The maximum difference between the 2.23 million group and the 3.62 million group is 7.16%; the maximum difference between the 3.62 million group and the 5.69 million group is 3.21%; and the maximum difference between the 5.69 million group and the 7.68 million group is the smallest, at only 1.64%. After the mesh independence test, it was found that the simulation results stabilized, starting from the third group of mesh numbers (i.e., when the number of mesh elements exceeded 3.62 million), and the differences between the results were minor. Considering the sharp increase in computing time caused by the massive number of grids, the simulation results with 5.69 million mesh elements were adopted in this case, to ensure computational efficiency. For lattice bone-repair scaffold models with similar structures, the number of mesh elements should be greater than 3 million.

It is worth noting that the CFD simulations performed in this study are primarily deterministic numerical models, meaning that the calculated hydrodynamic parameters represent exact mathematical solutions under the specified boundary conditions, rather than stochastic experimental data points. To ensure the numerical sensitivity and robustness of these deterministic results, the grid independence criteria established above guarantee that discretization errors are minimized. The subsequent comprehensive evaluations across multiple topologies and a broad porosity spectrum (40–70%) serve as a systematic parametric sweep, illustrating the continuous sensitivity of flow fields to geometric variations.

### 3.2. Model Validation

**Figure 7:**
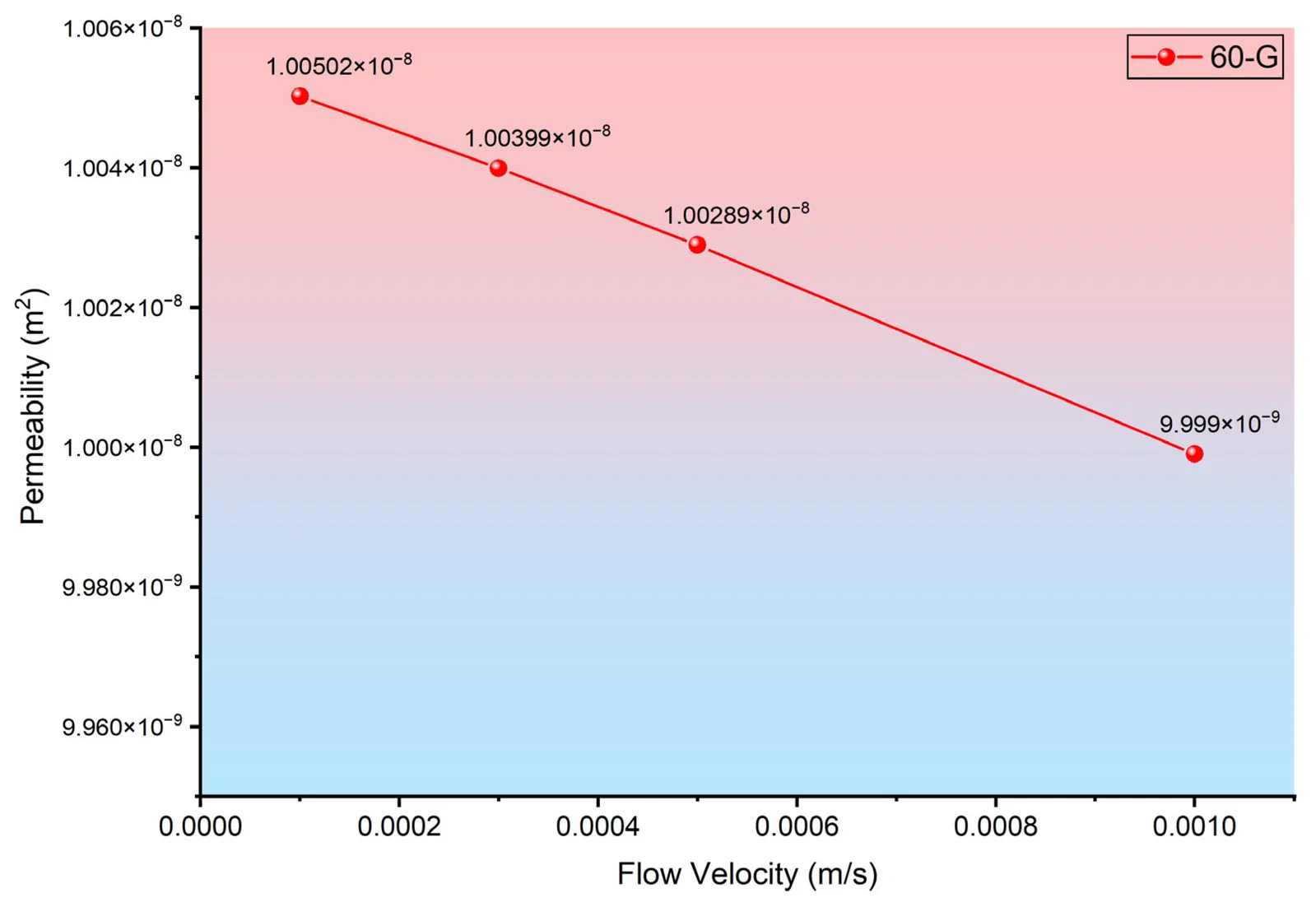
Permeability of scaffold at different flow velocities.

To verify the validity of the model, simulations were conducted on the 60-G scaffold at four different inlet velocities (1 mm/s, 0.5 mm/s, 0.3 mm/s, and 0.1 mm/s) while keeping all other conditions constant. The pressure drop generated by fluid flow through the scaffold under each velocity condition was extracted, and the scaffold permeability under different inlet velocities was calculated, based on Equation (1). The results are presented in Figure 7.

The results demonstrate that, under constant fluid properties, the scaffold permeability remains essentially independent of inlet velocity, with a maximum fluctuation of only 0.13%, which is consistent with the theoretical definition of permeability as an intrinsic material property in Darcy’s law. The minor fluctuations may originate from inlet/outlet boundary effects in the numerical simulation or from the computational precision limits of the solver.

To further validate the accuracy of the CFD model, the computed permeability of the 60-G scaffold across different inlet velocities was stable within the range of 1.0037 × 10^−9^ m^2^ to 1.0050 × 10^−9^ m^2^. Compared with the experimentally measured Darcian permeability of a Gyroid scaffold at the same porosity (60%) reported by Santos et al. [41] (1.195 × 10^−9^ m^2^), the relative deviation is 16.0–16.1%. This level of agreement is within the acceptable error range for CFD-experiment comparisons, and is primarily attributable to geometric deviations and surface roughness effects introduced during the 3D printing manufacturing process, consistent with numerical–experimental discrepancy patterns reported in the literature. Furthermore, the permeability values computed in this study (1.0037 × 10^−9^ m^2^ to 1.0050 × 10^−9^ m^2^) fall within the typical permeability range of natural trabecular bone (0.02 × 10^−9^ to 20 × 10^−9^ m^2^), further confirming the physiological plausibility of the model. Taken together, these multi-level validations demonstrate that the present CFD model accurately characterizes the hydrodynamic performance of bone-tissue engineering scaffolds.

### 3.3. Biomechanical Performance Analysis of Gyroid Lattice Scaffolds

**Figure 8:**
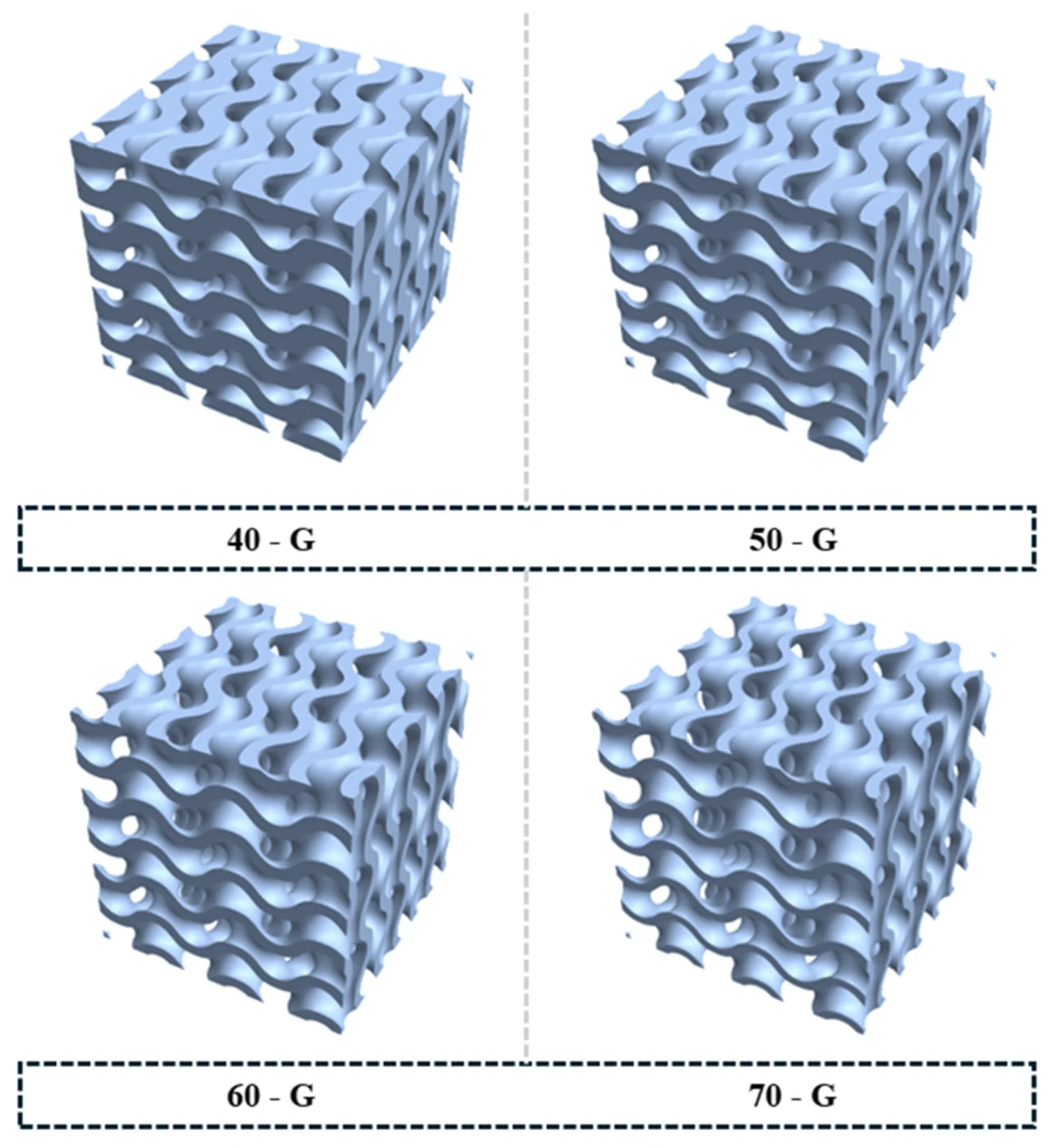
Models of Gyroid lattice scaffolds at different porosities.

The superficial layer of human bone is cortical bone, with a porosity of approximately 3–5%, while the interior is trabecular bone, with a porosity of 50–90% [42]. Porosity that is too low or too high will affect cell adhesion and growth; therefore, a porosity range of 40–60% was selected for research in this paper. Based on the Gyroid design, five groups of sheet-like TPMS structures with different porosities were designed, with porosities of 40%, 50%, 60%, and 70%, sequentially named 40-G, 50-G, 60-G, and 70-G. Each scaffold consists of 27 sheet-like Gyroid unit cells with a side length of 2 mm, and the models are uniformly arranged in a 3 × 3 × 3 format. The structure of each scaffold is shown in Figure 8 below. It is easy to see from the figure that as the porosity increases, the volume of the solid part of each sheet-like scaffold gradually decreases. The liquid material required for the simulation is DMEM, and the solid material is TC4. By summarizing the existing literature, the flow velocity typically set for bioreactors in various current simulation and laboratory experiments is 0.1 mm/s; therefore, the flow velocity was set to 0.1 mm/s in the simulation of this section. It is important to note that at this selected velocity, the flow is strictly within the creeping flow regime (Re = 0.0211, far below the critical threshold). Under these low-Reynolds-number conditions, the fluid inertial forces are negligible, and the governing Navier–Stokes equations behave linearly. Consequently, key hydrodynamic responses, such as pressure drop and WSS, scale proportionately with the inlet velocity. Therefore, while absolute values may vary under higher physiological flow rates or different bioreactor configurations, the relative performance trends and the comparative architectural advantages identified in this study remain robust and mathematically valid. Considering gravity, the boundary condition settings are consistent with the cases introduced in the previous chapter, and the same simulation parameter settings are adopted for each scaffold.

#### 3.3.1. Pressure Distribution

**Figure 9:**
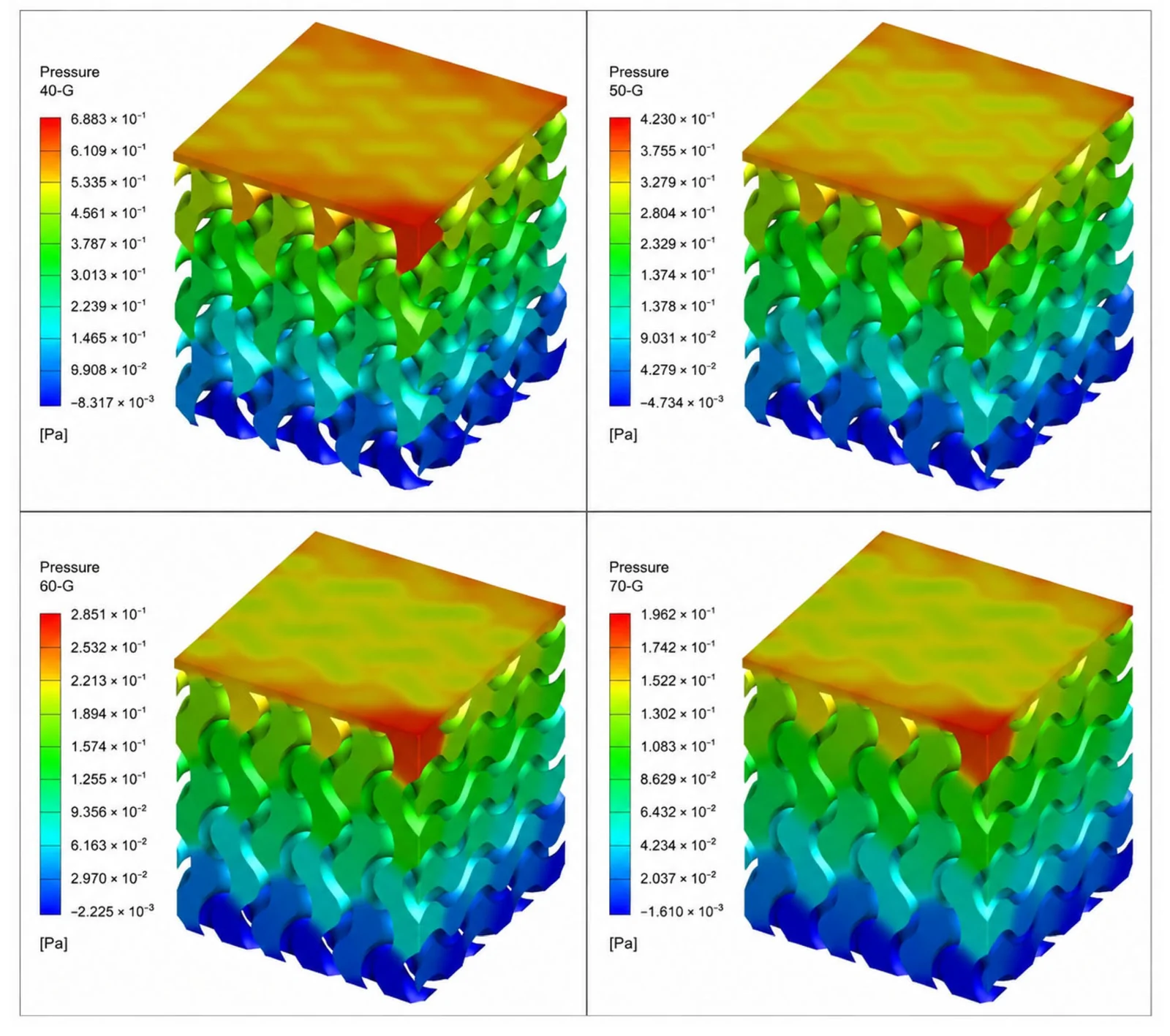
Pressure distribution of Gyroid lattice scaffolds at different porosities.

The pressure distribution of each scaffold is shown in Figure 9. As the porosity of the Gyroid scaffold increases from 40% to 70%, the pressure distribution within the fluid domain changes accordingly. For 40-G, the pressure ranges from a maximum of 6.883 × 10^−1^ Pa to a minimum of −8.317 × 10^−3^ Pa, exhibiting a large pressure gradient. In contrast, in the 70-G with a higher porosity, the maximum pressure is 1.962 × 10^−1^ Pa and the minimum is −1.610 × 10^−3^ Pa, showing a significantly narrowed pressure gradient. This trend indicates that the increase in porosity reduces the resistance to fluid flow, causing the pressure gradient to gradually flatten. According to the pressure distribution law in the contour maps, the pressure under each porosity decreases uniformly along the fluid flow direction, decreasing layer by layer from the inlet at z = 6.2 to the outlet at z = 0. However, at high porosities, the magnitude of the pressure drop is smaller, and the intensity of the negative pressure region is significantly weakened. This is because the increase in porosity expands the cross-section of the flow channels, reducing flow blockage, thereby making the pressure distribution more uniform and alleviating local vortices or flow separation phenomena. Further analysis reveals that when the porosity of the Gyroid sheet-like lattice scaffold increases from 40% to 70%, the maximum pressure value decreases by approximately 71.5%, while the absolute value of the minimum pressure decreases by approximately 80.6%. This variation trend confirms the optimizing effect of porosity on flow characteristics: higher porosity weakens the local flow resistance and reduces the overall pressure drop by expanding the effective area of the flow channels, indicating that regulating porosity can effectively balance the pressure drop and flow efficiency.

#### 3.3.2. Exit Area and Average Flow Velocity

**Figure 10:**
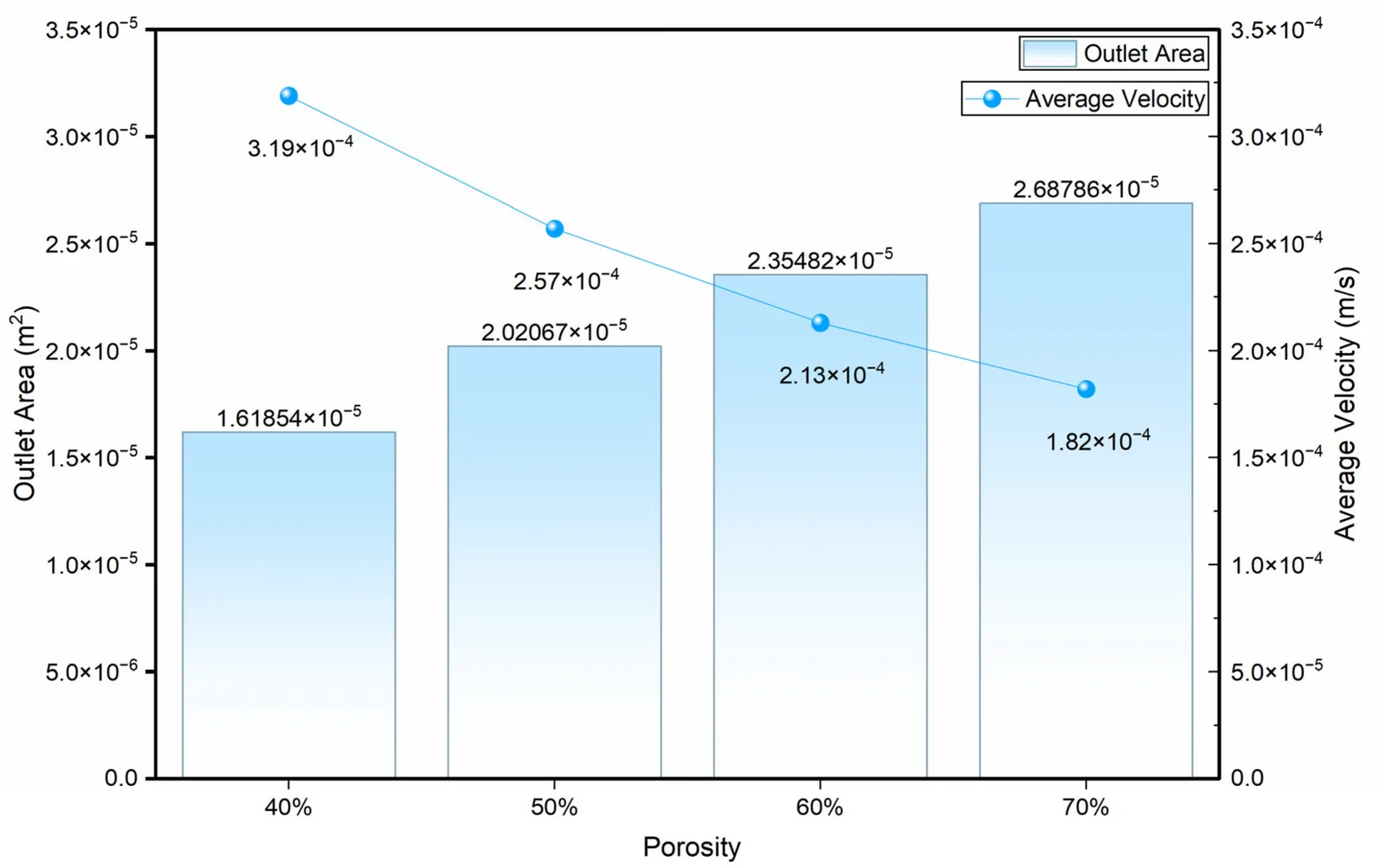
Changes in fluid outlet area and average velocity with porosity for different scaffolds.

As the porosity increases, the solid volume of the scaffold decreases, accompanied by a reduction in the wall thickness, which consequently leads to an increase in the fluid inlet and outlet areas. The fluid outlet areas corresponding to different porosities are shown in Figure 10. At a porosity of 40%, the Gyroid scaffold exhibits the minimum fluid outlet area, of only 0.00001685 m^2^. As the porosity increases, the outlet area gradually expands to 0.00002688 m^2^ at 70% porosity. It can be observed that for every 10% increase in porosity, the corresponding fluid outlet area increases by an average of approximately 18.5%, with the outlet area of the 70-G scaffold being 66.1% larger than that of the 40-G. Meanwhile, the increase in porosity also leads to a decrease in the average fluid velocity, as illustrated by the line chart in Figure 10. Due to its smallest outlet area, the fluid flow channels in 40-G are relatively narrow, resulting in the highest average velocity, with a peak value of 0.0003 m/s. With the enhancement of porosity, this value decreases to 0.00018 m/s for the 70-G scaffold, representing a reduction of 42.9%. This indicates that the increase in porosity effectively expands the flow channels within the scaffold, thereby causing the internal fluid velocity to become more uniform and steady.

#### 3.3.3. Velocity Distribution

**Figure 11:**
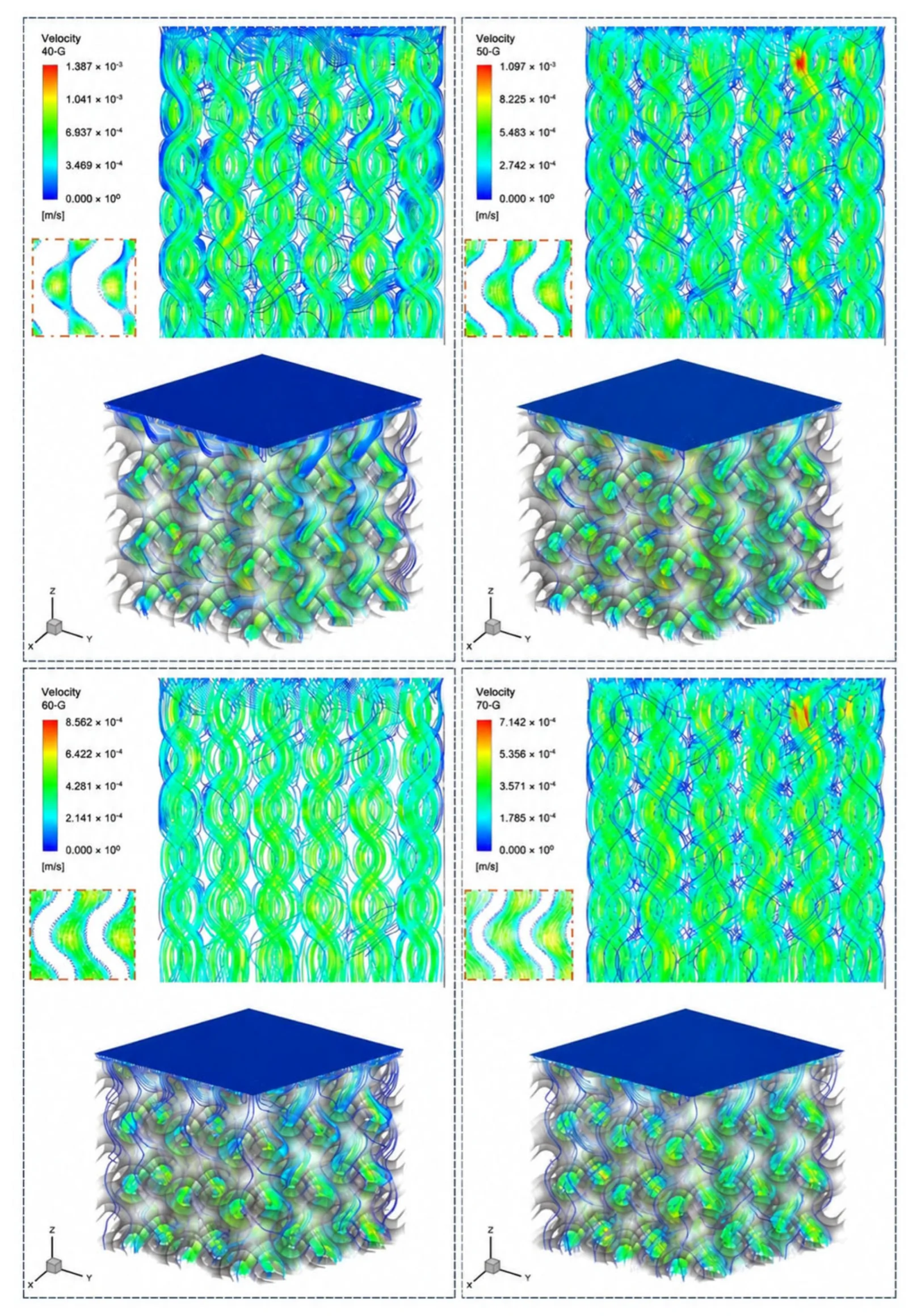
Velocity streamlines in Gyroid scaffolds at different porosities.

Figure 11 presents the velocity profiles created using 5000 streamlines, illustrating the fluid flow within the scaffolds. The bottom-left corner of the figure provides a detailed view of the velocity distribution inside the unit cells (where the white regions represent the solid structure of the scaffold), and the bottom part shows the corresponding isometric views of each scaffold. In this section, flow analysis was performed at a constant inlet velocity. It can be observed from Figure 11 that the flow velocity decreases as the porosity increases, with a reduction of 16–21% for every 10% increase in porosity. The results show that as the porosity of the Gyroid scaffold progressively increases from 40% to 70%, the maximum velocity within a single unit cell exhibits a relatively uniform downward trend. For 40-G, the maximum velocity is 1.387 × 10^−3^ m/s; it drops to 1.097 × 10^−3^ m/s for 50-G (a reduction of approximately 21%), further decreases to 8.562 × 10^−4^ m/s for 60-G (a reduction of approximately 22%), and reaches a minimum of 7.142 × 10^−4^ m/s for 70-G, which is 48.5% lower than that of 40-G. In low-porosity structures, the fluid is forced through narrow channels, leading to increased velocities, due to the Venturi effect. Conversely, high-porosity structures feature wider channels, resulting in a more uniform velocity distribution and reduced local velocity extremes, due to channel broadening. Although permeability increases, the maximum velocity may decrease because of this channel expansion. The velocity cross-sections reveal that the velocity distribution for all porosities decreases progressively from the center toward the edges, until reaching a stagnant state. This phenomenon is more clearly observed in the isometric views, where high-speed streamlines are concentrated in the central channels of the unit cells, while velocities are lower near the walls at the periphery. Consequently, the low-speed streamlines (blue) in the isometric views of all scaffolds are primarily concentrated near the surrounding walls. Among the scaffolds, 40-G exhibits the steepest velocity gradient, whereas the velocity attenuation in 70-G is relatively gradual, indicating improved flow-field uniformity at higher porosities. The decrease in velocity also corresponds to the reduced pressure drop shown in the pressure contour maps.

#### 3.3.4. Pressure Drop, Permeability, and Specific Surface Area

After calculation, the results of the inlet area, pressure drop, permeability, and specific surface area for each scaffold are summarized in Table 1, where the permeability and specific surface area were calculated according to Equations (1) and (2), respectively. The results show that as the porosity of the Gyroid scaffold increases from 40% to 70%, the inlet area gradually expands from 1.5918 × 10^−5^ m^2^ to 2.6885 × 10^−5^ m^2^. This indicates that higher porosity significantly expands the initial cross-sectional area of the fluid channels, which is consistent with the flattening of the pressure gradient in high-porosity scaffolds observed in the pressure contour maps, confirming the weakening effect of channel openness on flow resistance. The pressure drop (ΔP) decreases from 0.5552 Pa to 0.1473 Pa, a reduction of 73.5%, reflecting a positive correlation between porosity and permeability (with permeability increasing from 3.9175 × 10^−9^ m^2^ to 14.7681 × 10^−9^ m^2^). This suggests that high porosity significantly enhances the overall fluid transport capacity by improving channel connectivity. This trend in pressure drop is consistent with the experimental findings of Santos et al. regarding the pressure drop performance at different porosities when Re < 1. It further explains the impact of porosity on flow efficiency shown in the velocity maps: although the average velocity decreases due to channel dispersion, the increase in global permeability still dominates the optimization of fluid transport performance.

#### 3.3.5. WSS Distribution

**Figure 12:**
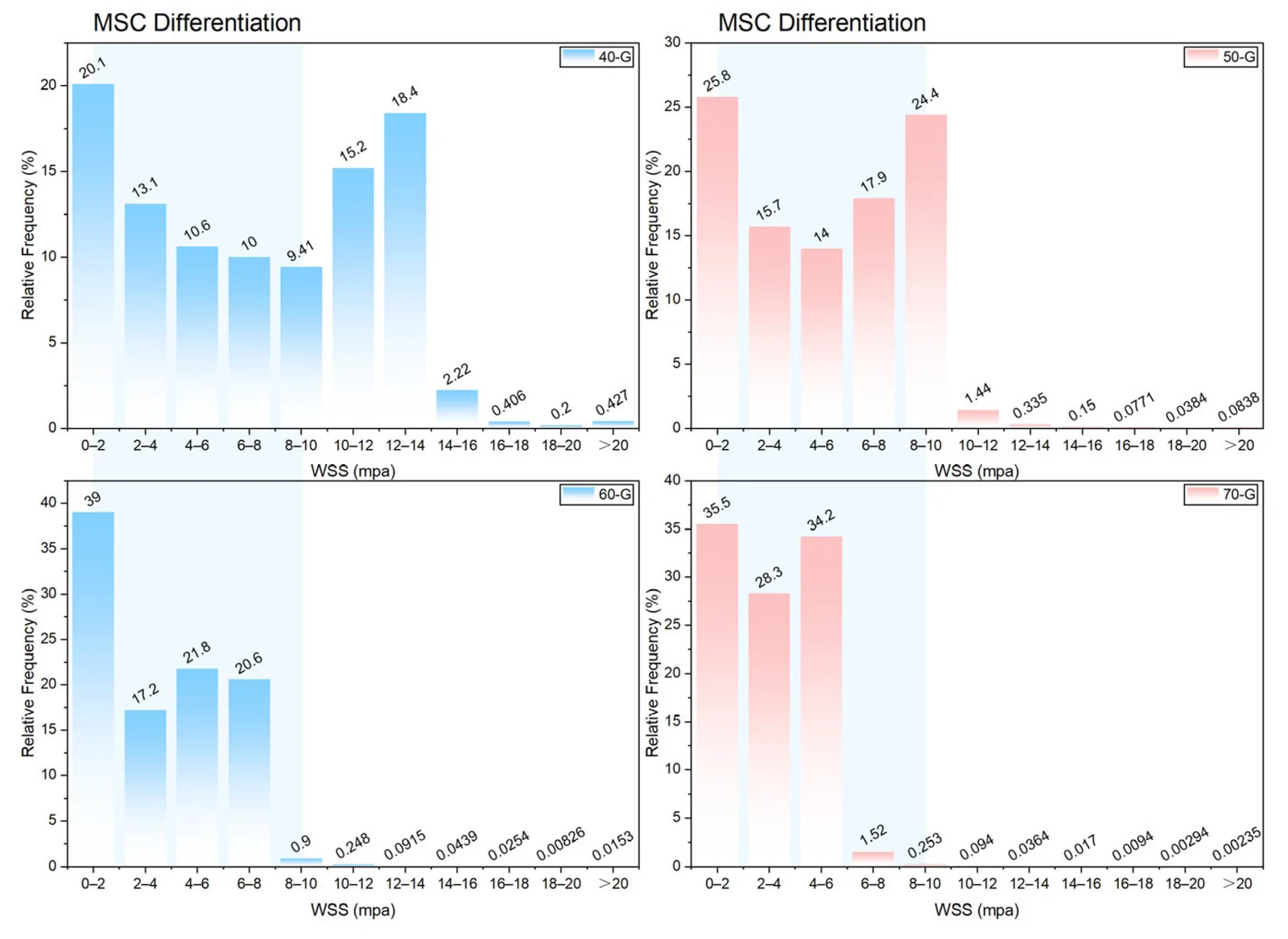
WSS distribution for different scaffolds.

Figure 12 illustrates the WSS distribution across different scaffolds. The results indicate that the wall shear stress on the surfaces of all Gyroid scaffolds is primarily concentrated within the range of 0–20 mPa. Specifically, for the 40-G scaffold, the cumulative proportion within the critical range for initiating mesenchymal stem cell (MSC) differentiation (0.1–10 mPa) reaches 82.8%, whereas this proportion increases to 85.7% for the 70-G scaffold. This suggest that increased porosity significantly expands the effective stress regions suitable for osteocyte adhesion and differentiation by optimizing channel connectivity. Meanwhile, the relative frequency of the 70-G scaffold in the 10–20 mPa range (0.773%) is nearly double that of the 40-G scaffold (0.406%), indicating that the enhancement of local fluid kinetic energy under high porosity can better promote the response of osteogenic precursor cells. Notably, the peak WSS for all scaffolds is controlled below 20 mPa (with the proportion in the >20 mPa range being <0.1%), thereby avoiding the risk of cell detachment caused by supra-physiological stress. This is consistent with the recommended WSS safety threshold for bone-tissue engineering (<15 mPa) found in the literature [31]. Furthermore, the relative frequency of the high-porosity scaffold (e.g., 70-G) in the 5–10 mPa range (25.8%) is significantly higher than that of the low-porosity scaffold (17.2%), verifying the positive regulatory effect of increased porosity on the osteogenic microenvironment. Combined with the analysis of pressure contour maps and velocity profiles, although high-porosity scaffolds exhibit a lower overall pressure drop and more dispersed velocity distribution, the increased coverage of WSS within key biomechanical ranges demonstrates that the synergistic design of porosity and channel topology can balance flow efficiency with the requirements for cellular mechanical stimulation.

### 3.4. Comparison of Gyroid- and Diamond-Lattice Scaffold Performance

**Figure 13:**
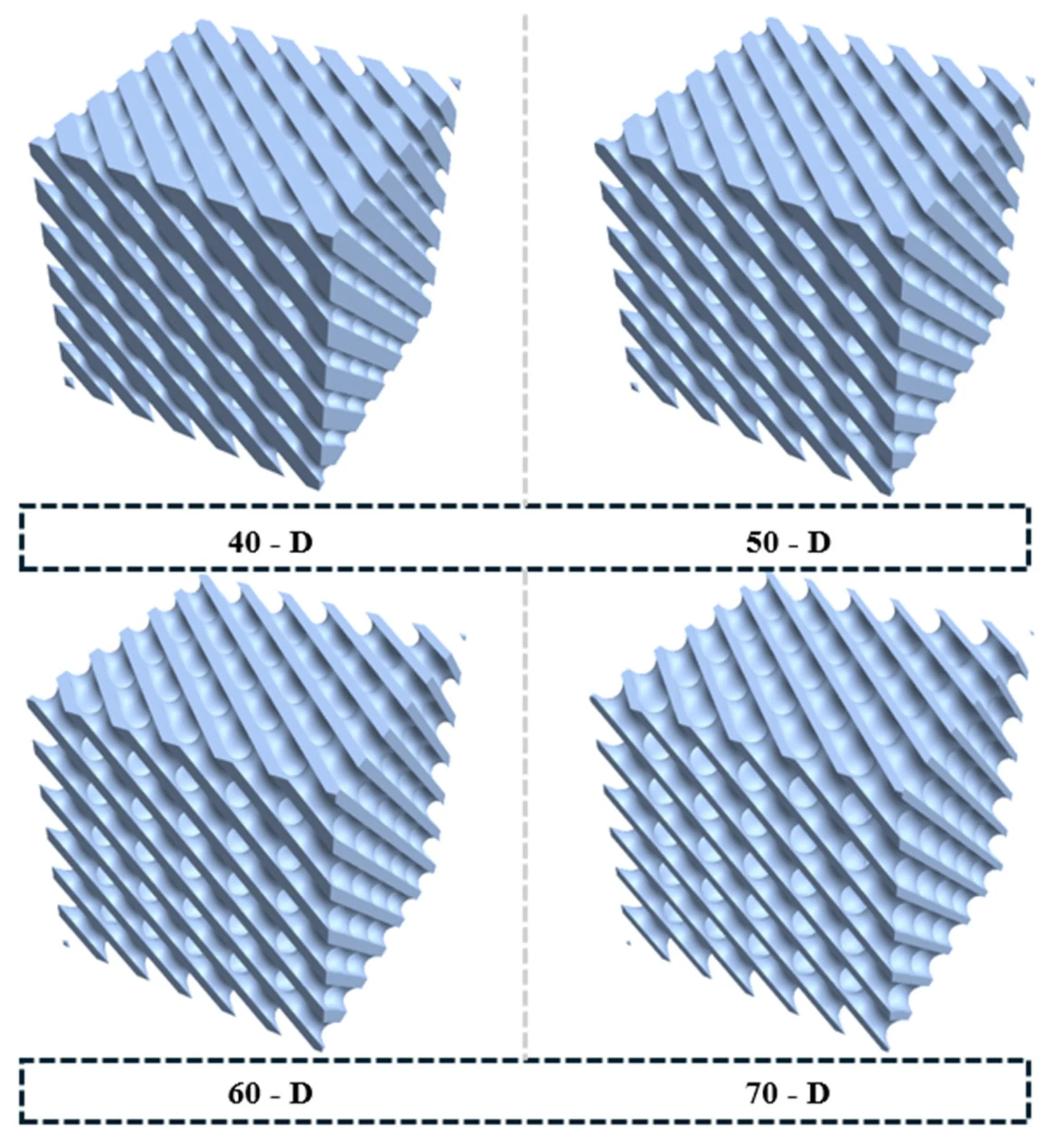
Diamond lattice scaffold models at different porosities.

Based on the established performance trends of the Gyroid structures, this section introduces the Diamond configuration and conducts a systematic comparison within the same porosity range, to reveal the characteristic differences between different topological architectures. The Diamond lattice scaffold models employed for simulation in this section are illustrated in Figure 13 below:

#### 3.4.1. Pressure Distribution

**Figure 14:**
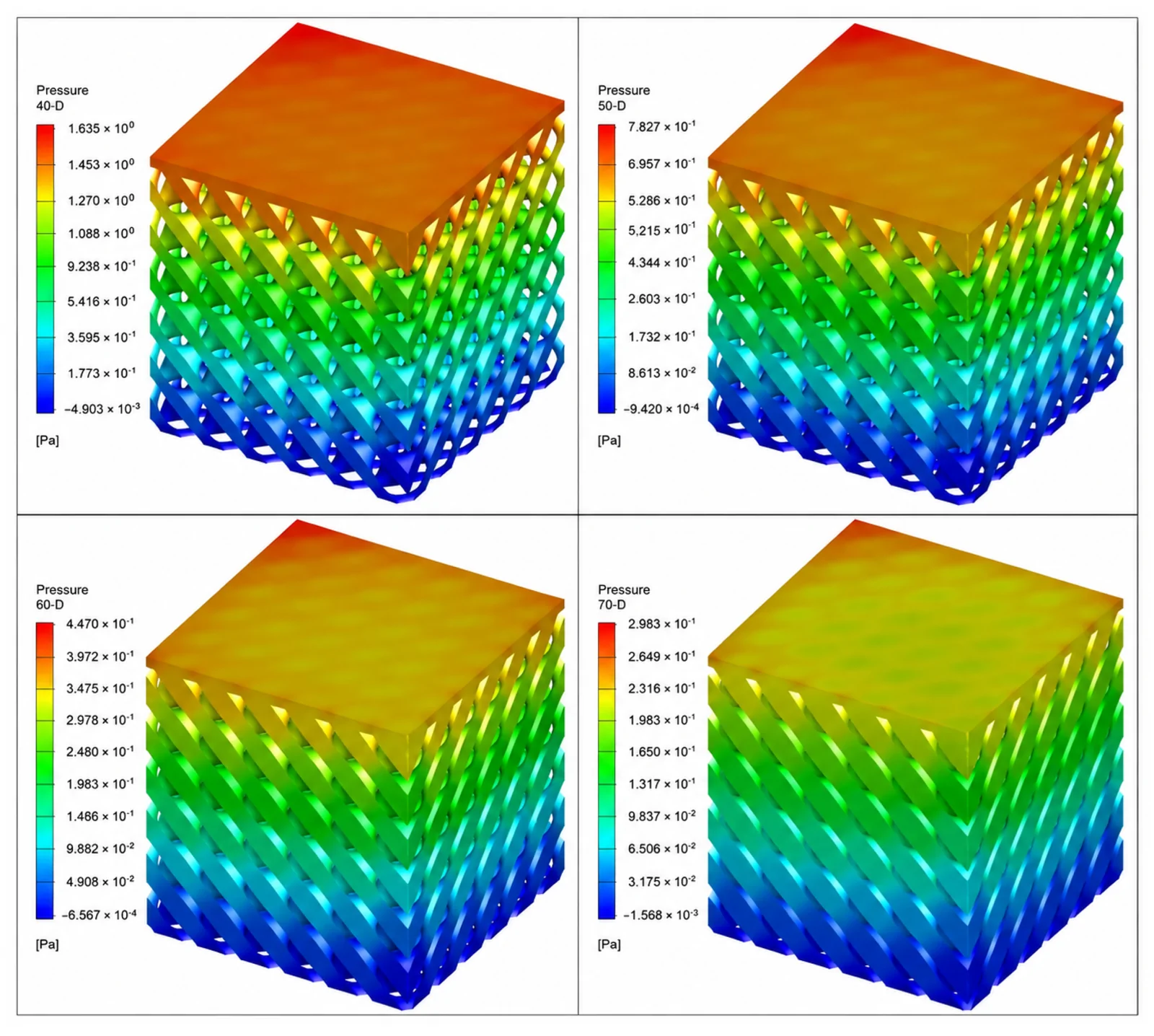
Pressure distribution for Diamond lattice scaffolds at different porosities.

The pressure contour maps obtained from the Diamond scaffold simulations are shown in Figure 14. The data reveal that as the porosity of the Diamond scaffold increases from 40% to 70%, the maximum pressure decreases significantly, from 1.635 Pa to 0.298 Pa, a reduction of 81.7%. Furthermore, the attenuation rate of the pressure gradient from the inlet to the outlet gradually slows down, indicating that the increase in porosity reduces flow resistance by expanding the cross-sectional area of the flow channels, which is consistent with the pressure-drop optimization trend observed in Gyroid scaffolds. Notably, a negative minimum pressure of −1.568 × 10^−3^ Pa appears in the 70-D scaffold, which may be attributed to the occurrence of slight backflow or vortex phenomena within its local flow channels. In comparison, the Gyroid scaffold (e.g., 70-G) exhibits a higher negative pressure intensity (−1.610 × 10^−3^ Pa) at the same porosity; this is also consistent at 40%, 50%, and 60% porosities. This reflects the fact that the curved-channel topology of the Gyroid structure may induce stronger local backflow or vortices, due to more complex fluid disturbances, whereas the channel structure of the Diamond scaffold relatively limits the intensity of flow separation.

Compared to Gyroid scaffolds, Diamond scaffolds consistently exhibit a higher pressure drop at the same porosity—for instance, the pressure drop of 60-D is 0.447 Pa, while that of 60-G is 0.216 Pa—indicating weaker channel connectivity in the Diamond structure. Furthermore, as porosity increases, the pressure distribution in Diamond scaffolds shows a marked increase in longitudinal asymmetry; for example, the 70-D pressure contour map presents a distinct longitudinal gradient. In contrast, Gyroid scaffolds maintain a more uniform pressure distribution, due to their isotropic curved-channel design. This highlights the differences in aerodynamic response between the two types of TPMS lattices, suggesting that the Gyroid type possesses superior advantages in optimizing flow efficiency and reducing energy dissipation.

#### 3.4.2. Exit Area and Average Flow Velocity

**Figure 15:**
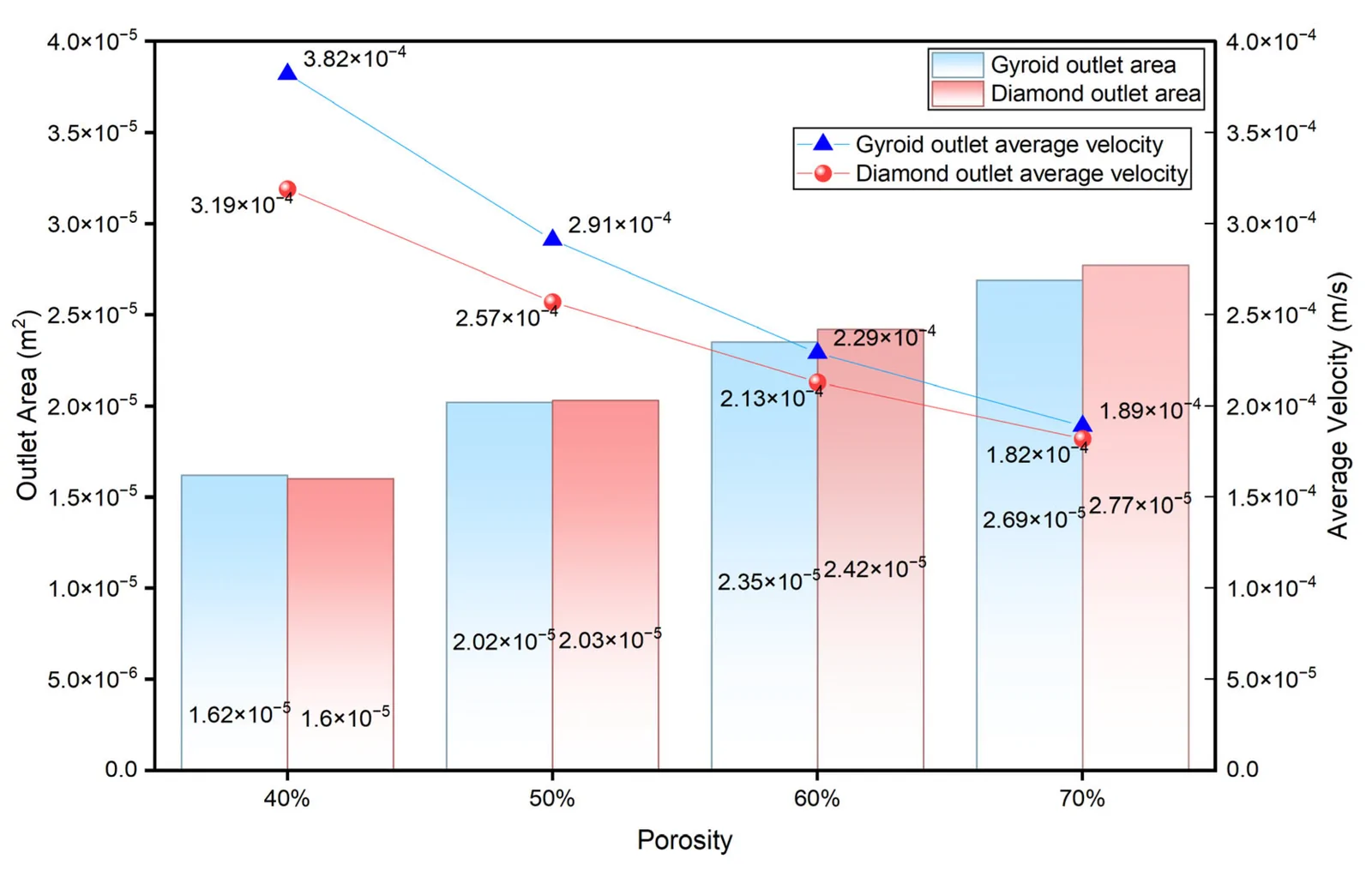
Outlet area and average flow velocity for Diamond scaffolds at different porosities.

The data for the outlet area and average fluid velocity of the Diamond scaffolds in Figure 15 show that as the porosity increases from 40% to 70%, the outlet area gradually expands from 1.60 × 10^−5^ m^2^ to 2.77 × 10^−5^ m^2^, representing an increase of 73.1%. Concurrently, the average velocity decreases from 2.02 × 10^−4^ m/s to1.82 × 10^−4^ m/s, a reduction of 9.9%. This phenomenon indicates that the increase in porosity weakens flow resistance by expanding the cross-sectional area of the flow channels; however, the velocity reduction is significantly lower than that of the Gyroid type (where the velocity of 70-G drops by 42.9%), reflecting the superior capacity of the Diamond channel topology to maintain flow velocity. Comparing the two scaffolds at 60% porosity, the outlet area of the Diamond structure is 1.2% smaller than that of the Gyroid type. Starting from 50% porosity, the fluid outlet area of the Diamond scaffold begins to exceed that of the Gyroid scaffold, with the 70-D outlet area (2.77 × 10^−5^ m^2^) being 2.97% higher than that of 70-G (2.69 × 10^−5^ m^2^). The average velocity of the Diamond scaffold is generally lower than that of the Gyroid scaffold, with a maximum difference of 19.7% at 60% porosity. This gap subsequently narrows to 3.85% at 70% porosity, where the velocities of 70-D and 70-G are 1.82 × 10^−4^ m/s and 1.89 × 10^−4^ m/s, respectively. The structural characteristics of the Diamond scaffold result in a lower channel tortuosity, compared to the Gyroid scaffold. By reducing tortuosity, the local kinetic energy is enhanced, whereas the average velocity in Gyroid scaffolds attenuates more significantly, due to channel dispersion. Furthermore, while the growth rate of the outlet area for the Diamond scaffold (73.1%) is slightly higher than that of the Gyroid (66%) as porosity increases, its velocity reduction is only about one-fourth of the latter, demonstrating the unique advantage of the Diamond structure in balancing channel expansion with kinetic energy preservation.

#### 3.4.3. Velocity Distribution

**Figure 16:**
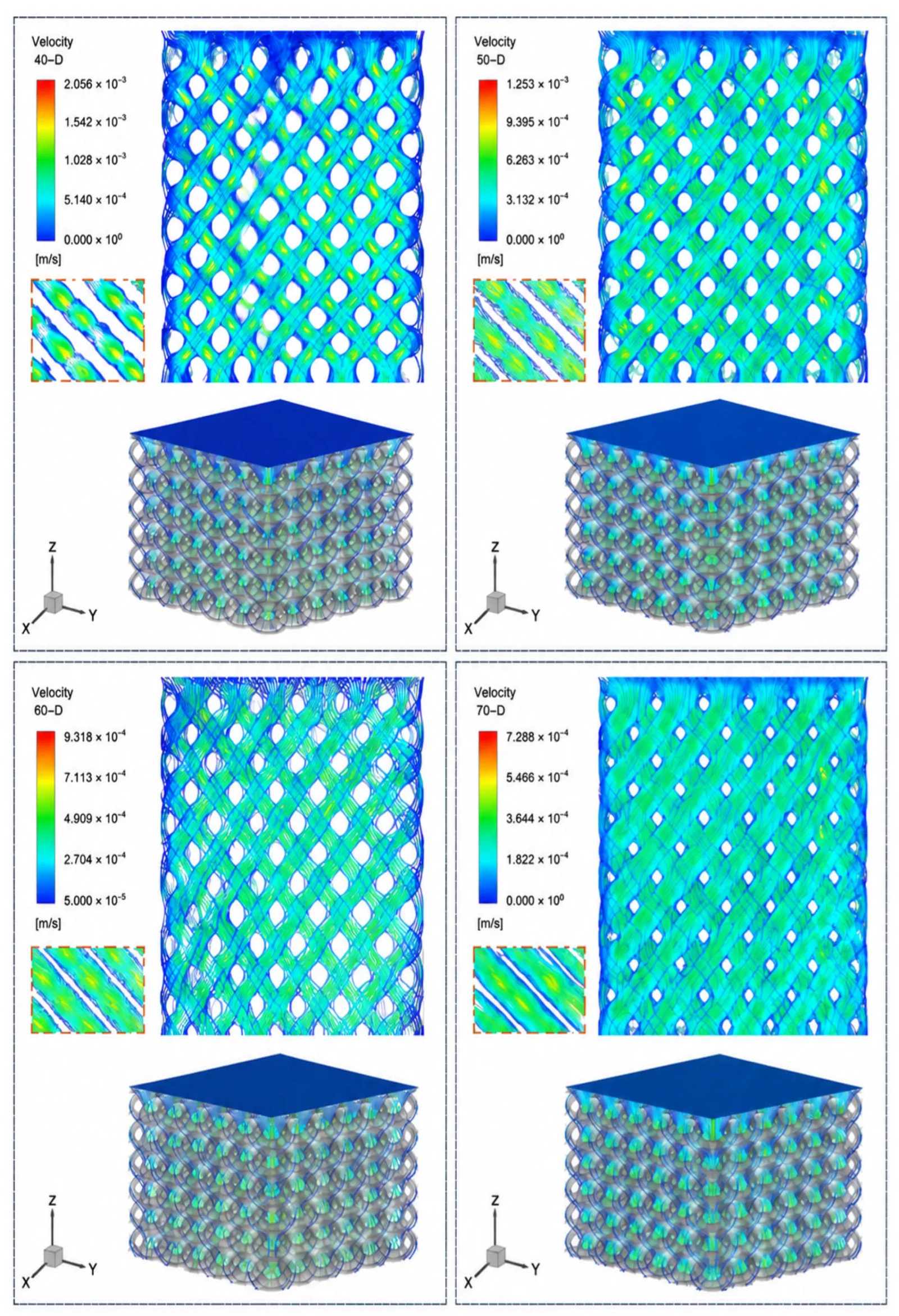
The streamlines of the fluid flow in different Diamond scaffolds.

Figure 16 displays the velocity streamlines for different Diamond scaffolds. In each velocity streamline plot, 5000 streamlines were generated from the inlet, consistent with the previous section. The main view illustrates the fluid flow velocity within the scaffold, while the bottom-left inset provides a detailed view of the flow within a single Diamond unit cell. The bottom part shows the isometric view of the entire scaffold, reflecting the macroscopic flow characteristics. As shown in Figure 16, the velocity distribution results for the Diamond scaffolds indicate an overall downward trend in internal flow velocity as porosity increases. The maximum velocity within the Diamond scaffolds decreases from 2.056 × 10^−3^ m/s to 7.288 × 10^−4^ m/s, representing a reduction of 64.5%. Compared to the Gyroid scaffolds in the previous section, this reduction is significantly higher than the 48.5% observed for the Gyroid type. A possible reason for this phenomenon lies in the structural characteristics of the Diamond configuration: at low porosities, the unique angular channels of the Diamond scaffold cause the fluid entering from the inlet to reach a higher initial velocity after passing through narrow passages. For instance, the maximum velocity in the 40-D scaffold is nearly 1.5 times that of the 40-G. However, as porosity increases, the expansion of the Diamond channel cross-sectional area leads to a more drastic attenuation of the maximum velocity, compared to the Gyroid. When comparing the two types, the maximum velocity of the Diamond scaffold remains slightly higher than that of the Gyroid at the same porosity; for example, the maximum velocity of 70-D is 7.288 × 10^−4^ m/s, while that of 70-G is 7.142 × 10^−4^ m/s.

Regarding the streamline distribution, both Diamond and Gyroid scaffolds exhibit similar general patterns: high-speed streamlines are concentrated in the internal channels of the lattice, while streamlines near the peripheral walls are sparse, and predominantly low-speed. This feature is clearly observable in the isometric views of all scaffolds, where streamlines near the walls are mostly deep blue (low-speed), whereas those in the internal channels are relatively concentrated, and light green. However, differences in flow details emerge between the two lattice types. Due to its structural characteristics, the Diamond velocity distribution exhibits a stronger “centralization” feature—main views of the Diamond scaffolds show high-speed streamlines concentrated along the axial regions of the angular channels, while the low-velocity regions near the walls account for a larger proportion compared to the Gyroid (indicated by the blue areas near the walls in the main views). For example, the low-velocity region in 70-D accounts for 62%, which is higher than the 55% in 70-G. This characteristic reflects the localized energy dissipation in Diamond channels caused by their angular geometry; specifically, as fluid passes through the angular channels, geometric variations induce turbulence and viscous shear in localized areas, leading to high energy dissipation. This property is also supported by the data in Figure 15: because the high-speed streamlines in the axial regions of the angles dominate the overall flow efficiency, the average velocity of the scaffold is maintained, despite the higher proportion of low-velocity wall regions. This “high-velocity core + low-velocity boundary” flow characteristic explains why, although the outlet area of the Diamond scaffold increases by 73.1% (higher than the 66.1% of the Gyroid) as porosity increases from 40% to 70%, the average velocity only decreases by 9.9% (significantly lower than the 42.9% of the Gyroid). In contrast, the curved channels of the Gyroid structure uniformize energy dissipation, leading to a more pronounced reduction in velocity.

#### 3.4.4. Pressure Drop, Permeability, and Specific Surface Area

Table 2 summarizes the results of the inlet area, pressure drop, permeability, and specific surface area for both the Gyroid and Diamond scaffolds. The data for the Diamond scaffold indicate that as porosity increases, the inlet area expands from 1.6099 × 10^−5^ m^2^ to 2.7634 × 10^−5^ m^2^, representing an increase of 71.6%, which is slightly higher than the 69.0% observed for the Gyroid scaffold. However, its reduction in pressure drop (decreasing from 1.4596 Pa to 0.2267 Pa, a drop of 84.5%) is significantly larger than that of the Gyroid scaffold (73.5%). With increasing porosity, the permeability of the Diamond scaffold rises from 1.4901 × 10^−9^ m^2^ to 9.5946 × 10^−9^ m^2^, achieving a total increase of 544%, while its specific surface area grows from 5.61 mm^2^/mm^3^ to 13.37 mm^2^/mm^3^, an increase of 138%. This reflects the fact that the synergistic optimization of porosity and surface area distribution can significantly enhance fluid transport capacity and cell attachment potential.

Nevertheless, across all porosity levels, the permeability of the Diamond scaffold remains consistently lower than that of the corresponding Gyroid scaffold, though their relative gap continuously narrows with increasing porosity. This relative discrepancy is most pronounced at 40% porosity, where the permeability of the Gyroid scaffold (3.9175 × 10^−9^ m^2^) is 163% higher than that of the Diamond scaffold (1.4901 × 10^−9^ m^2^). As porosity increases, this margin of superiority progressively shrinks to 102% (at 50% porosity), 70% (at 60% porosity), and 54% (at 70% porosity). This trend demonstrates that the concentrated flow resistance effect within Diamond channels is particularly prominent under low-porosity and narrow-channel conditions; however, as the channel cross-sections expand, the permeabilities of the two topologies gradually converge. This topology-dependent permeability behavior is in excellent agreement with the experimental findings of Castro et al. [43]. In physical flow tests at the same porosity (70%), they discovered that the permeability of the Gyroid scaffold was 49% higher than that of the Diamond scaffold, explicitly attributing this difference to the higher tortuosity and localized flow resistance inherent to the Diamond channel geometry, contrasted with the isotropic nature of the Gyroid curved channels. The quantitative agreement between the present CFD predictions and their experimental data further demonstrates that, under identical porosity conditions, the topological characteristics of the channels (isotropic vs. concentrated flow resistance) are the primary determinants of the permeability divergence between the two TPMS topologies, rather than the porosity itself. Furthermore, Castro et al. proved that this permeability ranking remains stable across different flow rates, further confirming that the observed differences stem from intrinsic structural properties, rather than flow regime-dependent effects.

In contrast, across all tested porosity levels, the specific surface area of the Diamond structure is consistently higher than that of the Gyroid structure at equivalent porosity (e.g., 13.37 mm^2^/mm^3^ versus 10.70 mm^2^/mm^3^ at 70% porosity, representing a 25% difference), thereby providing more attachment area for cells within the same volume. These distinctly contrasting quantitative-performance characteristics reveal a fundamental trade-off inherent in topological design: the Gyroid structure excels in fluid transport efficiency, owing to its isotropic channel network, whereas the Diamond structure compensates with a superior surface area for cell adhesion. Consequently, the selection between these two topologies should be tailored to specific application scenarios, guided by whether permeability or specific surface area serves as the primary design criterion in the target bone-repair strategy.

Table 2 summarizes the results for the inlet area, pressure drop, permeability, and specific surface area of both the Gyroid and Diamond scaffolds. The data for the Diamond scaffolds indicate that as porosity increases, the inlet area expands from 1.6099 × 10^−5^ m^2^ to 2.7634 × 10^−5^ m^2^, representing a 71.6% increase, which is slightly higher than the 69.0% observed in the Gyroid type. However, its 84.5% reduction in pressure drop (from 1.4596 Pa to 0.2267 Pa) is significantly greater than the 73.5% reduction for the Gyroid. With the increase in porosity, the permeability of the Diamond scaffold rises from 1.4901 × 10^−9^ m^2^ to 9.5946 × 10^−9^ m^2^, a total increase of 544%, while the specific surface area grows from 5.61 mm^2^/mm^3^ to 13.37 mm^2^/mm^3^, an increase of 138%. This reflects the fact that optimizing porosity and surface area distribution significantly enhances both fluid transport capacity and cell adhesion potential.

Nevertheless, the permeability of the Diamond scaffold at each porosity level remains consistently lower than that of the corresponding Gyroid type. For instance, at 70% porosity, the permeability of the Diamond scaffold is 9.5946 × 10^−9^ m^2^, whereas that of the Gyroid scaffold is 14.7681 × 10^−9^ m^2^, a difference of 53.9%. This is attributed to the channel characteristics of the Diamond structure, which lead to concentrated flow resistance. In contrast, the curved channels of the Gyroid structure reduce energy dissipation through an isotropic distribution, which is related to the isotropic nature of TPMS curved channels and results in superior channel connectivity compared to the Diamond structure—a conclusion consistent with the research by Castro et al. [43]. However, the Diamond structure possesses a higher specific surface area than the Gyroid structure at the same porosity, providing more cell attachment area within the same volume. These performance differences highlight the dual impact of topological design, and confirm the differentiated performance of the two scaffolds under flow-structure coupling.

#### 3.4.5. WSS Distribution

**Figure 17:**
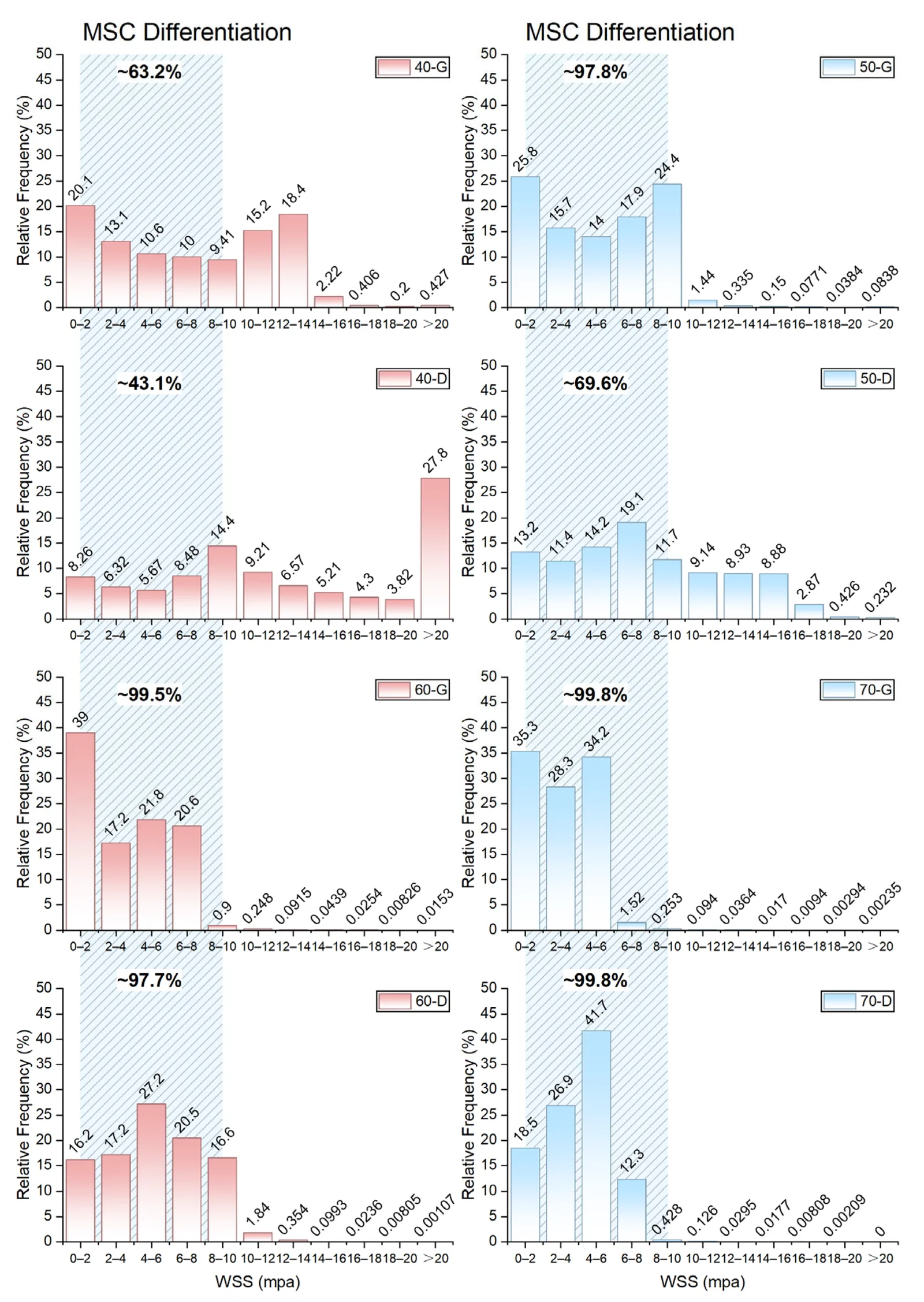
WSS distribution for different lattice-type scaffolds.

Figure 17 illustrates the WSS distribution statistics for the Diamond scaffolds. To analyze the differences between the Diamond and Gyroid lattice types, the WSS distribution results for both are integrated into the figure below. The data show that the wall shear stress (WSS) distribution on the surface of Diamond scaffolds exhibits non-uniformity. Specifically, the sum of the relative frequencies within the MSC differentiation range (0.1–10 mPa) for the 40-D scaffold is 43.1%, meaning that 43.1% of the scaffold surface area falls within the range suitable for MSC differentiation. However, 27.8% of the surface area of the 40-D scaffold also falls within the >20 mPa range, which may be attributed to the WSS concentration caused by the narrow flow channels of the Diamond scaffold under low-porosity conditions. As porosity increases, the proportion of the Diamond scaffold surface area exceeding 20 mPa decreases significantly, while the surface area within the MSC differentiation range gradually rises, reaching 69.6% for 50-D and 97.7% for 60-D. By 70% porosity, nearly the entire surface of the scaffold is suitable for MSC differentiation (99.8%). This trend indicates that with increasing porosity, the Diamond scaffolds successfully optimize WSS distribution through channel expansion; the relative frequency of local high WSS regions (>20 mPa) decreases markedly from 27.8% in 40-D to 0.2% in 70-D.

In contrast, the cumulative proportion of the Gyroid scaffold 40-G in the 0–10 mPa range is 63.2%, indicating that the Gyroid scaffold can provide a larger surface area suitable for cell proliferation than the Diamond scaffold under low-porosity conditions. Furthermore, the proportion of high WSS regions in the 40-G scaffold is only 0.427%, which is significantly lower than that of the Diamond scaffold. The curved channels of the Gyroid structure, leveraging their isotropic characteristics, uniformize energy distribution and reduce local stress concentration, thereby avoiding the potential risk of cell detachment. At high porosities of 60% and 70%, both Diamond and Gyroid scaffolds exhibit excellent WSS performance, with the vast majority of the surface area falling within the suitable range recommended in the literature. Notably, compared to the Gyroid scaffold, the stress distribution of the Diamond scaffold is more sensitive to changes in porosity. As porosity increases from 40% to 50%, the surface area of the Diamond scaffold within the suitable cell differentiation range increases by approximately 61.5%; further increasing the porosity to 60% results in a 40.4% increase in this area. After reaching 60% porosity, the WSS distribution of the Diamond scaffold tends to stabilize, with only a 2.1% increase in the suitable surface area as porosity grows further, suggesting that further increasing porosity has a limited regulatory effect on WSS under high-porosity conditions. For the Gyroid scaffold, the surface area in the MSC differentiation region increases by approximately 54.7% as porosity rises from 40% to 50%, with a mere 1.7% increase for 60-G compared to 50-G, and a 2.1% increase for 70-G compared to 60-G. Comparing the results, it is evident that the WSS of the Diamond scaffold is more sensitive to porosity changes under low-porosity conditions, while the Gyroid scaffold maintains a more uniform WSS distribution and achieves suitable performance at lower porosities, reflecting the characteristic differences between the two lattice types.

### 3.5. Porosity Gradient Design for Scaffold-Performance Optimization

Based on the aforementioned findings, we further explored whether a porosity gradient design could integrate the advantages of both architectures or optimize their respective inherent limitations. In this section, functionally graded Gyroid (40–60 G) and Diamond (40–60 D) scaffolds were developed, featuring a porosity transition ranging from 40% to 60%.

#### 3.5.1. Pressure Distribution

**Figure 18:**
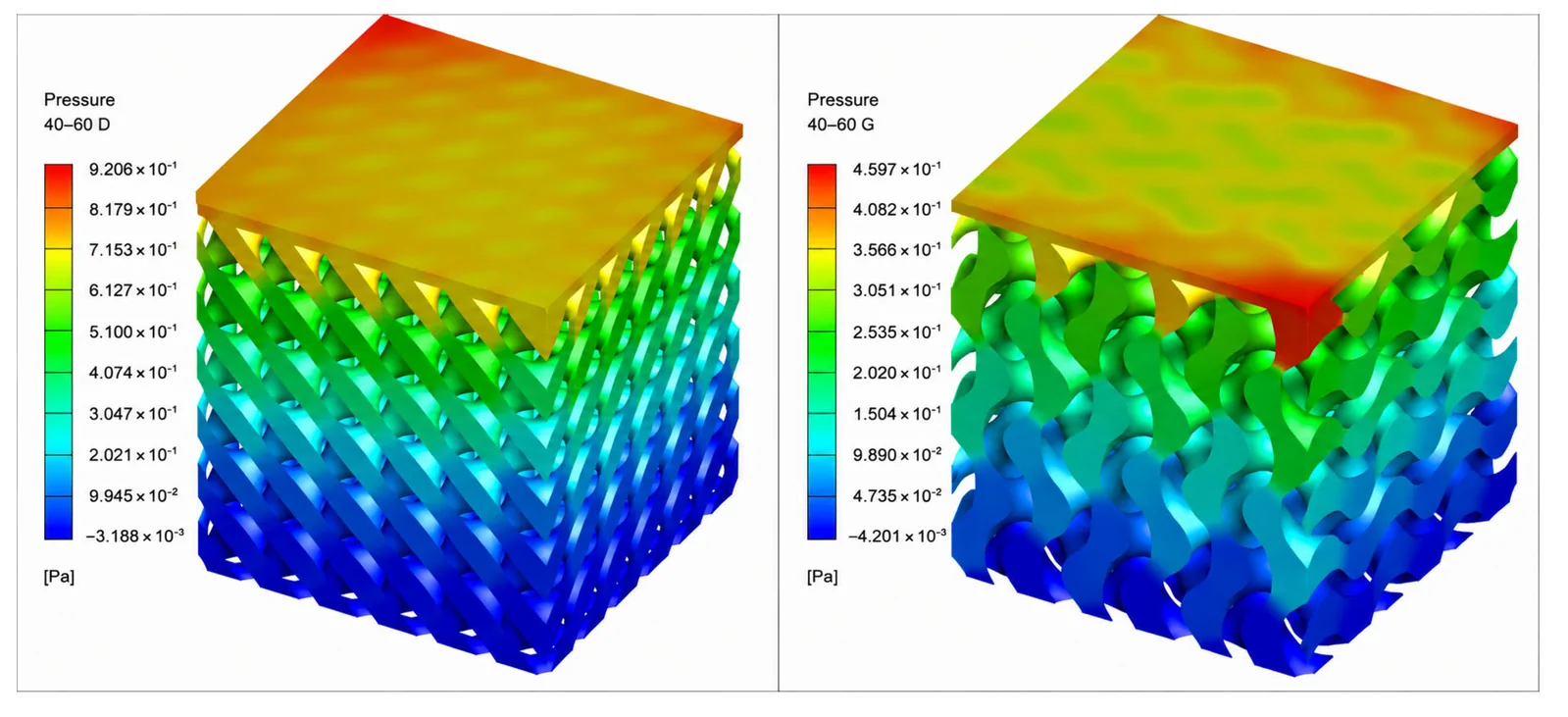
Pressure distribution for gradient and constant-porosity scaffolds.

Figure 18 illustrates the pressure distribution of the porosity-graded scaffolds (40–60 G and 40–60 D). For reference, the constant-porosity results of 40-G, 60-G, 40-D, and 60-D are presented in Figure 9 and Figure 14, respectively. The results for the graded scaffolds (40–60 D and 40–60 G) reveal a spatial dynamic transition in the pressure gradient, with pressure values decreasing sequentially. High-pressure regions are primarily concentrated at the fluid inlet, and as the fluid progresses along the flow direction, the pressure exhibits a descending trend, resulting in a graded stratification of the overall pressure field. Since the grading strategy involves an increase in porosity from 40% to 60% along the negative *z*-axis, the flow channel area expands progressively in the flow direction, facilitating smooth fluid transport through the scaffold interior. The pressure contours show an expanded low-pressure zone and a contracted high-pressure zone, indicating that the porosity gradient effectively disperses flow resistance through the incremental expansion of the channel cross-section, thereby avoiding localized pressure concentrations.

The peak pressure for the 40–60 Diamond scaffold is 9.206 × 10^−1^ Pa, situated between the 1.635 Pa of the constant 40-D and the 4.470 × 10^−1^ Pa of the 60-D. Meanwhile, the peak pressure for the 40–60 Gyroid is 4.597 × 10^−1^ Pa, significantly lower than the 6.883 × 10^−1^ Pa of the 40-G and approaching the 2.851 × 10^−1^ Pa of the 60-G. Its pressure contour also exhibits a uniform decline along the *z*-axis, ranging from 4.597 × 10^−1^ Pa to −4.201 × 10^−3^ Pa. Notably, the intensity of its negative pressure region is reduced by nearly 50%, compared to the −8.317 × 10^−3^ Pa of the 40-G, reflecting how the synergy between the isotropic characteristics of curved channels and the porosity gradient further optimizes flow-field homogeneity.

It can be observed that for both lattice types, the porosity grading strategy induces a gradual longitudinal attenuation in pressure distribution. In contrast, constant-porosity scaffolds, such as the 40-G, exhibit a uniform but much steeper gradient. The pressure difference (ΔP) between the maximum and minimum values for the 40-G is 0.697 Pa, whereas for the 40–60 G, it is reduced to 0.464 Pa, demonstrating that the gradient design effectively mitigates localized pressure surges through continuous porosity transitions. Similarly, the ΔP for the 40–60 D scaffold (0.9238 Pa) is 28.4% lower than that of the 40-D (1.6399 Pa). Although this difference remains higher than that of the 60-D (0.4477 Pa), the longitudinal asymmetry in the 40–60 D is significantly weakened, particularly in the mid-section near Z = 3, where the gradient plateaus. This suggests that the porosity gradient effectively alleviates the geometric singularity effects associated with the Diamond’s angular channels.

Overall, the porosity grading strategy balances the low flow resistance of high-porosity regions with the high-pressure characteristics of low-porosity regions by dynamically modulating the channel cross-section. This achieves a lower overall pressure drop and a more uniform stress distribution, providing a stable mechanical microenvironment for cells. These results validate the efficacy of the porosity grading strategy in balancing channel expansion and energy dissipation, providing a critical foundation for the multifunctional optimization of biomimetic bone-repair scaffolds.

#### 3.5.2. Exit Area and Average Flow Velocity

**Figure 19:**
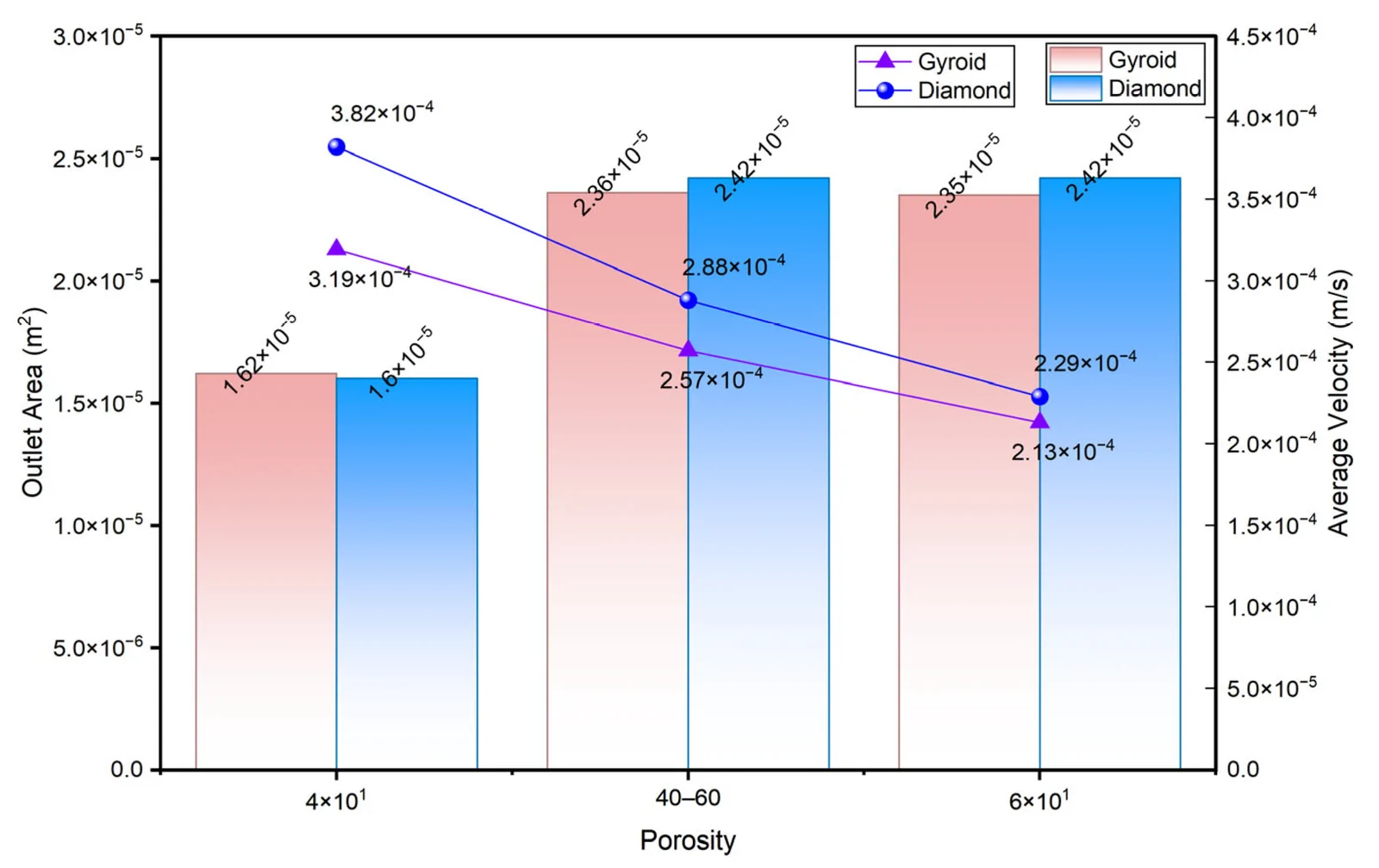
Outlet area and average flow velocity for gradient and constant-porosity scaffolds.

According to the data in Figure 19, the porosity-graded scaffolds demonstrate a significant advantage in outlet area compared to the constant-porosity models. Taking the 40–60% porosity gradient as an example, the outlet area of the Diamond scaffold is 2.42 × 10^−5^ m^2^, which is larger than that of the 40-G (1.6 × 10^−5^ m^2^) and matches the outlet area of the 60-D scaffold. Because the continuous variation in porosity optimizes the geometric distribution of fluid channels and mitigates localized flow blockage, the effective flow area of the scaffold is expanded. Consequently, the average velocity of the graded scaffolds—such as the 40–60 G at 2.57 × 10^−4^ m/s—is significantly lower than the 3.19 × 10^−4^ m/s observed in the constant-porosity 40-G scaffold, while remaining slightly higher than that of the 60-G. Furthermore, under the gradient design, the flow velocity in the Diamond architecture remains higher than in the Gyroid architecture (e.g., 2.88 × 10^−4^ m/s for 40–60 D vs. 2.57 × 10^−4^ m/s for 40–60 G). This suggests that within a porosity gradient, the Diamond structure may exhibit higher flow retention, due to the lower tortuosity of its unit-cell connectivity.

#### 3.5.3. Velocity Distribution

**Figure 20:**
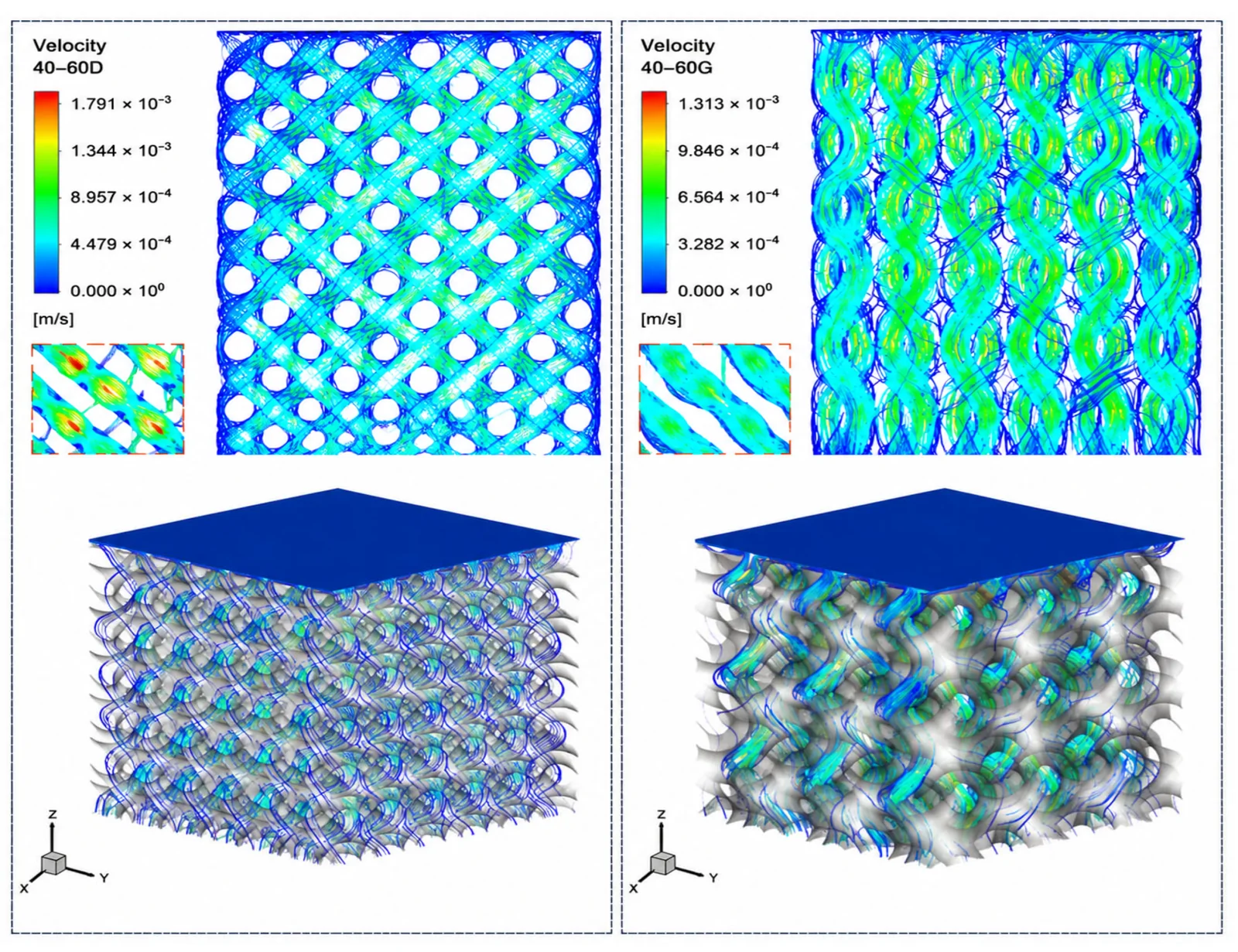
Velocity streamlines in porosity-graded and constant-porosity scaffolds.

Figure 20 illustrates the velocity streamlines within the porosity-graded scaffolds (40–60 G and 40–60 D). For reference, the constant-porosity results of 40-G, 60-G, 40-D, and 60-D are presented in Figure 11 and Figure 16, respectively. It is observed that in the graded architectures, the flow channels widen significantly along the direction of fluid flow, leading to a marked reduction in the maximum flow velocity. Specifically, the peak velocity of the 40–60 D scaffold decreases by approximately 12.9% compared to the 40-D model, while the maximum velocity of the 40–60 G scaffold shows a more modest reduction of 5.3%, relative to the 40-G model. As shown in the profiles, the Diamond architecture exhibits concentrated streamlines with higher velocities in the central regions, whereas the velocities near the peripheral walls are lower. In the 40–60 D scaffold, the progressive increase in porosity results in widened flow pathways and intensified velocity loss. Consequently, toward the outlet, low-velocity streamlines (indicated in blue) increasingly accumulate near the wall surfaces. This specific region is highly conducive to nutrient sequestration and promotes cell residence within the scaffold. Conversely, the porosity-graded Gyroid scaffold, benefiting from its extensive and continuous curved surfaces, achieves a more homogeneous velocity distribution throughout the structure, compared to the Diamond type.

#### 3.5.4. Pressure Drop, Permeability, and Specific Surface Area

Table 3 presents the optimized integrated performance of the 40–60% porosity-graded Diamond and Gyroid scaffolds, relative to their constant-porosity counterparts. Taking the 40–60-G as an example, its inlet area of 1.6106 × 10^−5^ m^2^ is slightly larger than that of the constant-40%-porosity scaffold (1.5918 × 10^−5^ m^2^). The pressure drop (0.3496 Pa) is lower than that of the 40-G model (0.5552 Pa), but remains higher than the 60-G model (0.2164 Pa), indicating that the grading strategy effectively reduces flow resistance while maintaining favorable fluid throughput. Furthermore, the permeability of the 40–60-G (6.2211 × 10^−9^ m^2^) exhibits a significant 37% increase over the 40-G (3.9175 × 10^−9^ m^2^), highlighting the potential of porosity gradients in enhancing transport capacity. The specific surface area (6.37 mm^2^/mm^3^) is higher than that of the 40-G (5.19 mm^2^/mm^3^), but lower than the 60-G (8.08 mm^2^/mm^3^), demonstrating an expansion of the material exchange interface while avoiding the structural fragility typically associated with high-porosity architectures. A similar trend is observed in the 40–60-D group: its pressure drop (0.7281 Pa) is substantially reduced compared to the 40-D (1.4596 Pa), its permeability (2.9874 × 10^−9^ m^2^) is double that of the 40-D (1.4901 × 10^−9^ m^2^), and its specific surface area (7.19 mm^2^/mm^3^) also outperforms the constant-40%-porosity group. Overall, the porosity grading strategy achieves an effective balance between pressure drop, permeability, and specific surface area by synergizing the characteristics of high- and low-porosity regions. This design not only improves fluid transport efficiency, but also reconciles structural stability with functional surface availability. Compared to single-porosity (40% or 60%) scaffolds, the graded design is better suited for application scenarios requiring the synergistic optimization of multiple performance metrics.

#### 3.5.5. WSS Distribution

**Figure 21:**
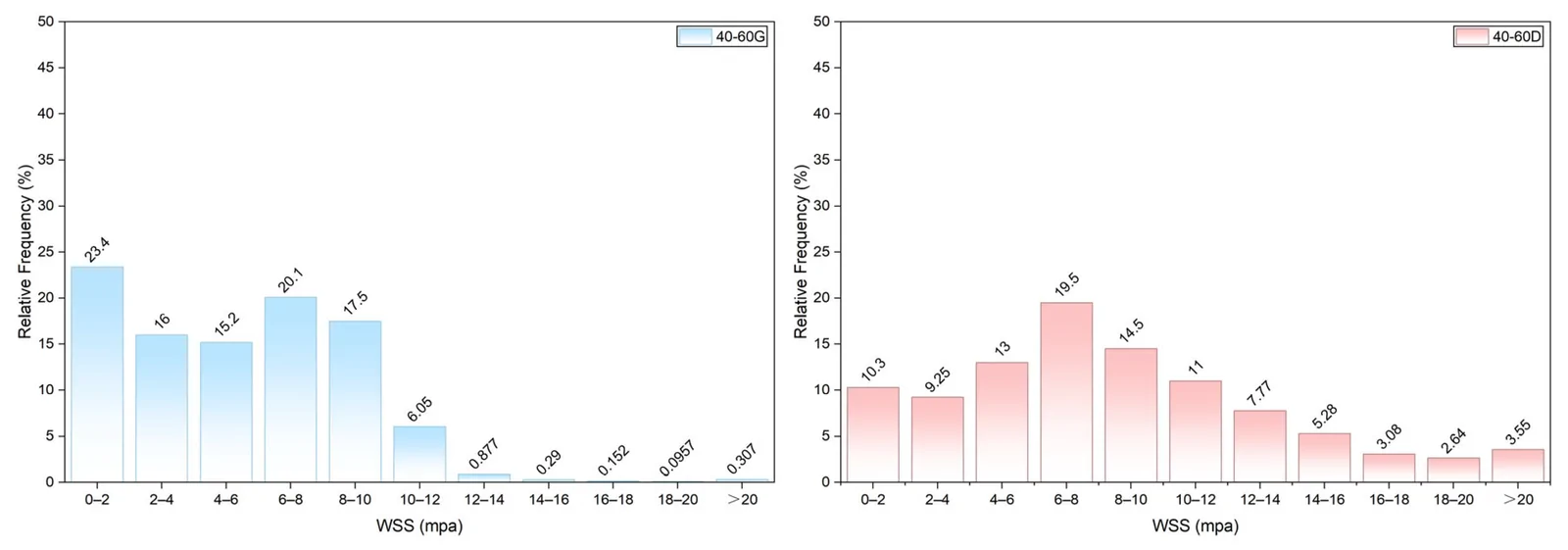
WSS distribution for gradient and constant-porosity scaffolds.

Figure 21 illustrates the WSS distribution across the porosity-graded scaffolds (40–60 G and 40–60 D). For reference, the constant-porosity results of 40-G, 60-G, 40-D, and 60-D are presented in Figure 17. It is evident from the profiles that, under the influence of the porosity grading strategy, the WSS distribution for all scaffold types converges toward the 0.1–10 mPa range, which is a critical window for mesenchymal stem cell (MSC) differentiation. The surface area of the 40–60 G scaffold within this differentiation zone rises to approximately 92.2%, representing a 45.9% increase in the area conducive to cell proliferation, compared to the constant 40-G model. Meanwhile, the 40–60 D group accounts for 66.6% of the surface area in this region—a 54.5% improvement over the 40-D model, although it remains slightly lower than the 60%-porosity control group. These findings demonstrate that the porosity grading strategy effectively optimizes the cellular-proliferation performance of the scaffolds.

## 4. Conclusions

This study focuses on the numerical simulation and functional design of porous bone-repair scaffolds, systematically exploring the construction strategies of triply periodic minimal surface (TPMS) lattice structures and their regulation mechanisms on biomechanical properties. Combined with functional gradient design concepts, an innovative method for optimizing the performance of bone-tissue engineering scaffolds is proposed. Through numerical simulation techniques, the dynamic effects of porosity and grading strategies on scaffold permeability, wall shear stress (WSS) distribution, and hydrodynamic characteristics are revealed, providing theoretical support for the design of biomimetic bone-repair scaffolds. The main research findings are as follows:

(1) The solid construction and performance advantages of sheet-like TPMS scaffolds were clarified. The study achieved the precise transformation from ideal curved surfaces to solid structures through level-set equations and isosurface offset strategies. The results indicate that the sheet-like structure exhibits excellent structural integrity and flow channel interconnectivity at low relative densities (5–30%). Compared with traditional structures, it possesses higher permeability in the low-density range and effectively alleviates the stress shielding effect during bone repair through a uniform stress distribution.
(2) A functional grading design strategy based on porosity gradients was proposed. To address the heterogeneous characteristics of natural bone, a dynamic porosity grading-scaffold scheme was designed. The study found that the porosity gradient scaffold (e.g., the 40–60% graded design), by dynamically adjusting the cross-sectional area of internal flow channels while ensuring mechanical support, reduced the pressure drop by 28.4% and increased permeability by 37%, compared to homogeneous scaffolds. Furthermore, this strategy significantly optimized the flow-field environment, increasing the coverage of wall shear stress (WSS) within the osteogenic differentiation range (0.1–10 mPa) by 45.9%.
(3) The regulation laws of porosity on the bio-hydrodynamic performance of sheet-like scaffolds were revealed. Numerical simulations showed that permeability exhibited a non-linear growth trend with increasing porosity (a 70% porosity increased permeability by up to 277%, compared to 40% porosity), while the pressure drop decreased significantly, by 73.5%. The study established the coupling relationship among “porosity–permeability–WSS distribution” and confirmed that the optimal balance between high permeability and suitable shear stress can be achieved by precisely adjusting the geometric parameters of the single-phase sheet structure.
(4) The influence of the level-set parameter *c* on scaffold performance and its optimization path were analyzed. The research found that although increasing the level-set constant *c* can enhance the mechanical stability of the scaffold by increasing wall thickness, it leads to the compression of the flow channel space, causing a significant drop in permeability. This indicates that in the customized design of scaffolds, it is necessary to optimize the gradient transition scheme of *c* to mitigate local performance losses, thereby achieving the dual optimization of mechanical adaptability and bio-fluid transport performance.
(5) A complete multi-software collaborative design and numerical simulation workflow was established. A full-process scheme covering mathematical modeling in Mathematica, geometric correction in Fusion, defect detection in Magics 24.0, fluid domain extraction in SpaceClaim, and, finally, high-precision CFD simulation in Fluent was proposed. This workflow promotes geometric fidelity and computational convergence during the numerical simulation of complex sheet-like scaffolds, providing reliable technical support for the research and development of biomimetic bone-repair scaffolds.

In summary, this study established a quantitative model of “porosity-lattice type-biomechanical performance” for TPMS lattice scaffolds through multi-scale hydrodynamic simulations, providing a key theoretical basis for the topological design and functional adaptation of biomimetic bone-repair scaffolds.

## 5. Future Directions

Future research should emphasize the integration of dynamic models for cell growth and mineralization. This study did not account for the dynamic impact of cell proliferation, migration, and mineralization processes on the scaffold’s pore architecture. In the future, by coupling cellular mechanics models with fluid dynamics simulations, a multi-physics prediction framework can be established to further enhance the biological accuracy of numerical simulations.

Furthermore, while the Diamond architecture exhibits notable theoretical manufacturing advantages—specifically, its favorable self-supporting topology that mitigates the need for internal sacrificial supports during additive manufacturing (AM)—practical fabrication limitations must be critically considered. When translating these idealized CAD models into physical metallic prototypes via laser powder bed fusion (LPBF), severe surface roughness typically arises from the staircase effect and the adherence of partially melted powder particles inside the tortuous micro-channels. From a hydrodynamic perspective, this elevated micro-roughness serves as localized flow resistance, which will inherently increase the overall pressure drop and trigger localized wall shear stress (WSS) spikes or micro-scale flow separations. Additionally, geometric deviations such as strut dimensional mismatch (thickening at nodal junctions or thinning of inclined struts) can cause noticeable shifts between the designed nominal porosity and the actual as-built porosity. While internal fabrication defects, such as micro-voids, predominantly dictate the mechanical fatigue tolerance, rather than the fluidic permeability, a comprehensive calibration utilizing micro-computed tomography (μ-CT) data to reconstruct real-surface CFD meshes remains a vital avenue for our ongoing research, to bridge the gap between deterministic numerical baselines and experimental realizations.

The purely numerical simulation results presented in this paper establish a crucial theoretical baseline, but they need to be further validated through additive manufacturing (e.g., SLM, EBM) and in vitro experiments (e.g., perfusion bioreactors). Specifically, future studies will incorporate experimental permeability tests on 3D-printed scaffolds to validate the CFD predictions. This is particularly important to investigate how practical manufacturing limitations—such as surface roughness, geometric deviations, and localized microporosity inherent to additive manufacturing—might alter the idealized hydrodynamic microenvironment characterized in this study. A closed-loop “design–simulation–manufacturing–testing” workflow could be established in the future to optimize the manufacturability of the scaffolds and calibrate model parameters. This will drive the translation of research findings into clinical applications and enhance the connection between manufacturing processes and performance verification.

While the current study focuses on hydrodynamic regulation, future work could combine mechanical stimulation (e.g., cyclic compressive loading) and biochemical factor release models. This would allow for the exploration of bone regeneration mechanisms under the synergistic action of mechanical, chemical, and thermodynamic factors, promoting the development of “smart-responsive” scaffolds and advancing the collaborative optimization of multimodal regulation strategies.

This paper primarily investigates single or gradient structures, whereas natural bone exhibits a multi-level heterogeneity characterized by a cortical-to-trabecular transition. Subsequent work could simulate the multi-scale characteristics of natural bone through spatial gradient combinations of lattice types, porosity, and material properties (e.g., an outer layer of high-density Diamond + an inner layer of high-porosity Gyroid). This approach would achieve functional synergy between mechanical support and nutrient transport, further extending and integrating the functional grading-scaffold design strategy proposed in this study to explore the combinatorial design of heterogeneous functional grading scaffolds.

Additionally, while this study focused on a linear 40–60% porosity gradient to establish a controlled comparison baseline, exploring alternative gradient bounds (such as 40–70% or 50–80%) and non-linear gradient functions (e.g., parabolic or exponential profiles) represents a compelling direction for further optimization. Shifting the gradient boundaries could help tailor the scaffold for specific anatomical sites with distinct load-bearing demands, though it requires careful management of the trade-off between high-porosity structural fragility and low-porosity shear stress concentrations. Furthermore, incorporating non-linear gradients could theoretically minimize localized hydrodynamic bottlenecks by smoothing fluid velocity transitions along the flow axis, thereby yielding a highly optimized and stable microenvironment for long-term cell culture.

In summary, this study provides a systematic approach for the biomimetic design of bone-tissue engineering scaffolds. Future interdisciplinary collaboration and technology integration are expected to overcome existing bottlenecks and drive the innovative development of personalized and functionalized bone-repair materials.

## Figures and Tables

**Table 1:** Inlet Area, Pressure Drop, Permeability, and Specific Surface Area for Different Scaffolds.

Scaffold	Inlet Area (×10^−5^ m^2^)	Pressure Drop (Pa)	Permeability (×10^−9^ m^2^)	Specific Surface Area (mm^2^/mm^3^)
40-G	1.5918	0.5552	3.9175	5.19
50-G	2.0223	0.3299	6.5938	6.38
60-G	2.3698	0.2164	10.0502	8.08
70-G	2.6885	0.1473	14.7681	10.70

**Table 2:** Summary of Inlet Area, Pressure Drop, Permeability, and Specific Surface Area for Gyroid and Diamond Scaffolds.

Scaffold	Inlet Area (×10^−5^ m^2^)	Pressure Drop (Pa)	Permeability (×10^−9^ m^2^)	Specific Surface Area (mm^2^/mm^3^)
40-G	1.5918	0.5552	3.9175	5.19
50-G	2.0223	0.3299	6.5938	6.38
60-G	2.3698	0.2164	10.0502	8.08
70-G	2.6885	0.1473	14.7681	10.70
40-D	1.6099	1.4596	1.4901	5.61
50-D	2.0320	0.6669	3.2611	7.14
60-D	2.4219	0.3678	5.9130	9.53
70-D	2.7634	0.2267	9.5946	13.37

**Table 3:** Summary of Inlet Area, Pressure Drop, Permeability, and Specific Surface Area for Gradient and Constant-Porosity Scaffolds.

Scaffold	Inlet Area (m^2^ × 10^−5^)	Pressure Drop (Pa)	Permeability (m^2^ × 10^−9^)	Specific Surface Area (mm^2^/mm^3^)
40-G	1.5918	0.5552	3.9175	5.19
40–60 G	1.6106	0.3496	6.2211	6.37
60-G	2.3698	0.2164	10.0502	8.08
40-D	1.6099	1.4596	1.4901	5.61
40–60 D	1.6063	0.7281	2.9874	7.19
60-D	2.4219	0.3678	5.9130	9.53

## Data Availability

The original contributions presented in this study are included in the article. Further inquiries can be directed to the corresponding author.
